# HIV-1 N-myristoylation-dependent hijacking of late endosomes/lysosomes to drive Gag assembly in macrophages

**DOI:** 10.1242/jcs.263588

**Published:** 2024-11-21

**Authors:** Gabriel I. Guajardo-Contreras, Ana L. Abdalla, Alex Chen, Meijuan Niu, Erwan Beauchamp, Luc G. Berthiaume, Alan W. Cochrane, Andrew J. Mouland

**Affiliations:** ^1^ Department of Medicine, McGill University, Montreal, QC H4A 3J1, Canada; ^2^ Lady Davis Institute at the Jewish General Hospital, Montreal, QC H3T 1E2, Canada; ^3^ Department of Microbiology and Immunology, McGill University, Montreal, QC H3A 2B4, Canada; ^4^ The Institute of Medical Sciences, University of Toronto, Toronto, ON M5S 1A8, Canada; ^5^ Department of Molecular Genetics, University of Toronto, Toronto, ON M5S 1A8, Canada; ^6^ Pacylex Pharmaceuticals Inc., Edmonton, AB T5J 4P6, Canada; ^7^ Department of Cell Biology, University of Alberta, Edmonton, AB T6G 2H7, Canada

**Keywords:** HIV-1, Late endosome, N-myristoylation, PCLX-001, Viral trafficking, Virus-containing compartment

## Abstract

Macrophages represent an important viral reservoir in HIV-1-infected individuals. Different from T cells, HIV-1 assembly in macrophages occurs at intracellular compartments termed virus-containing compartments (VCCs). Our previous research in HeLa cells – in which assembly resembles that found in infected T cells – suggested that late endosomes/lysosomes (LELs) play a role in HIV-1 trafficking towards its assembly sites. However, the role of LELs during assembly at VCCs is not fully understood. Herein, we used the HIV-1-inducible cell line THP-1 GagZip as a model to study HIV-1 Gag intracellular trafficking and assembly in macrophages. We demonstrated LEL involvement at VCCs using various microscopy techniques and biochemical approaches. Live-cell imaging revealed that HIV-1 repositions LELs towards the plasma membrane and modulates their motility. We showed that Arl8b-mediated LEL repositioning is not responsible for Gag trafficking to VCCs. Additionally, the inhibition of myristoylation by PCLX-001 decreased the presence of Gag on endosomes and inhibited VCC formation in both the THP-1 cell line and primary macrophages. In conclusion, we present evidence supporting the idea that HIV-1 manipulates the LEL trajectory to guide Gag to VCCs in an N-myristoylation-dependent manner.

## Teaser

**Summary:** HIV-1 Gag N-myristoylation is required for co-trafficking with late endosomes/lysosomes, altering their motility to drive virus assembly at virus-containing compartments in macrophages.

## INTRODUCTION

Currently, 39.9 million people are living with HIV-1, the etiological agent of acquired immunodeficiency syndrome, or AIDS (UNAIDS, https://www.unaids.org/en/resources/fact-sheet). Combinatorial antiretroviral therapy (cART) is used to inhibit HIV-1 replication, but fails to eliminate HIV-1 from latently infected cells (Henderson et al., 2020). HIV-1 productively infects CD4+ T cells and macrophages, where, in the latter, virus assembly and budding take place at intracellular compartments termed virus-containing compartments (herein referred to as VCCs), instead of the plasma membrane (Tan and Sattentau, 2013; Joshi et al., 2009). Additionally, macrophages can be latently infected, representing an important viral reservoir from where HIV-1 can rebound when cART is interrupted (Honeycutt et al., 2016, 2017; Arainga et al., 2017). VCCs are defined as plasma membrane invaginations that are transiently connected to the cell surface (Nkwe et al., 2016; Gaudin et al., 2013; Bennett et al., 2009). They play fundamental roles in cell-to-cell transmission, virus spread and immune evasion within the host (Lopez et al., 2019; Chu et al., 2012).

HIV-1 assembly is coordinated by the structural polyprotein Gag, which must traffic within the cytoplasm to make its way to the assembly sites (Freed, 2015). Our previous studies have shown that a subpopulation of Gag and its viral RNA co-traffics with late endosomes/lysosomes (LELs) in HeLa cells (Cinti et al., 2017; Lehmann et al., 2009). When LELs are repositioned to the plasma membrane or to juxtanuclear regions, a concomitant increase or decrease, respectively, in virus release is observed (Lehmann et al., 2009). Moreover, an HIV-1 variant lacking Gag N-terminal myristoylation abolishes the colocalization between Gag, the viral (v)RNA and LELs, supporting the hypothesis that Gag requires anchoring to LEL membranes to traffic within infected cells (Lehmann et al., 2009).

Recent research has shifted the perception of LELs from their being a disposal-related organelle to their being a highly dynamic signaling hub that extensively communicates with other cellular structures, redefining LELs as a regulatory hub for cellular homeostasis and an attractive therapeutic target for a broad variety of disease conditions (Ballabio and Bonifacino, 2020). In the context of HIV-1-infected macrophages, the membranes that are part of VCCs have been described as having a low abundance of LELs markers, meaning they are not LEL-derived compartments; however, they have been often observed in close proximity to each other (Deneka et al., 2007; Pelchen-Matthews et al., 2003). Whether LELs play any role in Gag trafficking towards VCCs for virus assembly remains an open question.

Herein, we evaluated the THP-1-derived cell line named THP-1 GagZip as a model for HIV-1 assembly in macrophages. This cell line was generated by transducing a doxycycline-inducible HIV-1 genome that encodes Gag fused to GFP as a reporter protein (Das et al., 2004; Zhou et al., 2006). We used this cell line to evaluate the relevance of LELs in HIV-1 Gag trafficking using various biochemical and microscopy approaches. VCCs in THP-1 GagZip macrophages resemble those formed following *in vitro* HIV-1 infection of monocyte-derived macrophages (MDMs) (Welsch et al., 2011, 2007; Gousset et al., 2008). Likewise, we confirmed the presence of LELs at VCCs by confocal microscopy, as well as the presence of Gag on LEL membranes by subcellular fractionation and detection of endosomal populations using flow cytometry. We then evaluated the co-existence of LELs with Gag near the plasma membrane by total internal reflection fluorescence (TIRF) microscopy, as well as LEL motility in macrophages using three-dimensional live-cell imaging. Our data suggest that HIV-1 repositions LELs to the plasma membrane, increasing their dwell time at this location. Interestingly, only LELs colocalizing with Gag showed altered motility, supporting the idea that HIV-1 commandeers and requires LELs to traffic towards VCCs. In parallel, we evaluated whether Arl8b-mediated LEL repositioning, or the inhibition of N-myristoyltransferases 1 and 2 (NMT1 and NMT2, collectively NMT1/2) activity, affected VCCs formation. Our results indicate that Arl8b antagonizes VCC formation, whereas the inhibition of NMT1/2 by the drug PCLX-001 (Zelenirstat) impaired HIV-1 assembly and VCC formation in both the THP-1 cell line and primary macrophages. These results highlight the relevance of LELs and Gag co-trafficking in macrophages, suggesting new pharmaceutical approaches for targeting the HIV-1 reservoir.

## RESULTS

### THP-1 GagZip-expressing macrophages exhibit a VCC phenotype

**Fig. 1.**
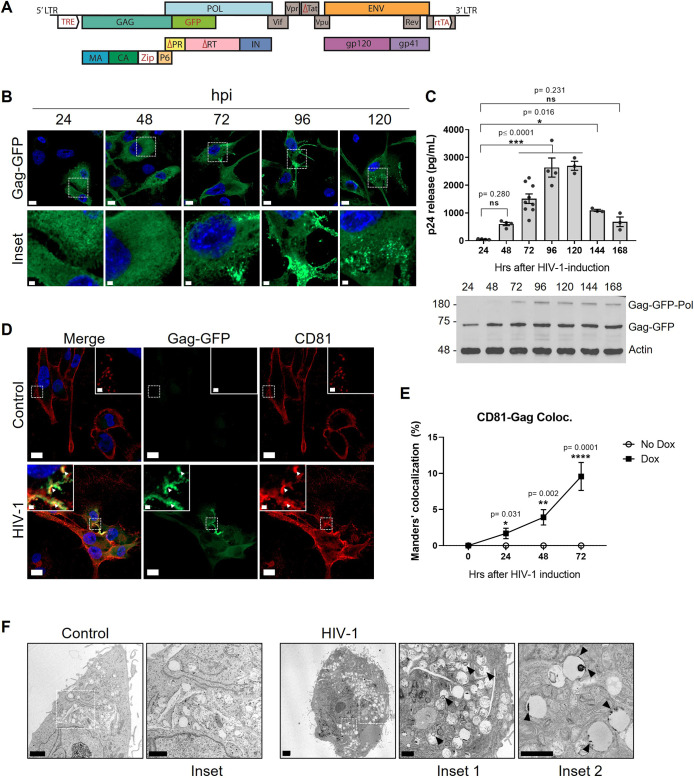
**THP-1 GagZip macrophages resemble VCCs phenotype 72 h after HIV-1 induction.** (A) Scheme of engineered HIV-1 genome integrated into the THP-1 GagZip cell line. The nucleocapsid domain within Gag is replaced by a leucine zipper domain (Zip), and Gag is in-frame fused with the reporter gene GFP. The HIV-1 promoter region and the accessory protein Nef were replaced by the tetracycline response element (TRE) and the reverse tetracycline transactivator (rtTA), respectively. Triangles (Δ) indicate viral genes with deletions to produce replication-incompetent viral particles. (B) Confocal microscopy from fixed THP-1 GagZip macrophages evaluated for Gag–GFP expression every 24 h after HIV-1 induction. Scale bars: 10 µm (top); 2 µm (bottom). (C) HIV-1 p24 release into the supernatant every 24 h after HIV-1 induction, measured by ELISA (top panel). Western blot from cell lysates collected every 24 h after HIV-1 induction (bottom panel), evaluated for Gag–GFP expression, using β-actin as a loading control. (D) Confocal microscopy from fixed THP-1 GagZip macrophages 72 h after HIV-1 induction or control conditions, stained by immunofluorescence to detect Gag–GFP and CD81. White arrowheads indicate colocalization between Gag–GFP (green) and CD81 (red) clusters. Scale bars: 15 µm (main images); 1 µm (inset). (E) From D. Bar graph representing Manders' percentage of colocalization of CD81 over Gag–GFP evaluated at 0, 24, 48 and 72 h after HIV-1 induction for control (No Dox) and HIV-1 induced (Dox) THP-1 GagZip macrophages. (F) Transmission electron microscopy from THP-1 GagZip macrophages 72 h after HIV-1 induction. Images from a non-induced macrophage are shown on the left side. Images from an HIV-1 induced macrophage are shown on the right side. Vacuolar structures filled with viral particles or protein aggregates at the luminal side are pointed with black arrowheads. Scale bars: 2 µm (main images); 1 µm (magnified insets). All data representative from three independent experiments. Graphs are presented as mean±s.e.m. Statistical analyses in C were performed by one-way ANOVA, using Dunnett's post hoc test to compare each data set against 24 h timepoint. In E, a two-way ANOVA test, using Sidak's post hoc test to compare each time-point between control versus HIV-1 induction conditions was used. *P<0.05; **P≤0.01; ***P≤0.001; ****P<0.0001; ns, not significant.

HIV-1 infection in macrophages has been widely studied *in vitro* by infecting MDMs obtained from peripheral blood mononuclear cells (PBMCs) from healthy donors (Nkwe et al., 2016; Gaudin et al., 2013; Bennett et al., 2009; Chu et al., 2012; Welsch et al., 2011, 2007; Gousset et al., 2008). MDMs are more refractory to HIV-1 infection compared to CD4+ T cells, making it challenging to observe VCCs (Cassol et al., 2006; Swingler et al., 2007; Yuan et al., 2017). To study VCCs in a robust model, we evaluated their formation using the human pro-monocytic cell line THP-1 GagZip. This cell line was transduced with an engineered version of the HIV-1 LAI strain genome, coding for the viral polyprotein Gag fused in frame to GFP at its C-terminus. Additionally, the HIV-1 promoter was mutated and replaced with the Tet-ON system (Fig. 1A), allowing for the induction of the HIV-1 DNA transcription in the presence of doxycycline (Zhou et al., 2006; Das et al., 2004). This system was used here to study the late stages of HIV-1 replication.

We first evaluated whether THP-1 GagZip macrophages accumulated HIV-1 particles at VCCs, as observed in human MDMs, by monitoring Gag–GFP expression and virus release every 24 h after HIV-1 induction. During the first 48 h, Gag–GFP was homogenously distributed in the cytoplasm, but at 72 h after induction, Gag–GFP accumulated at distinct areas near the plasma membrane (Fig. 1B). In parallel, as a measure of viral release, we quantified HIV-1 p24 in the supernatant from cultured macrophages by ELISA. We observed a time-dependent increase of p24 levels, reaching a peak at 96 h post-induction (2637±346 pg ml^−1^; mean±s.e.m.), followed by an ∼4-fold decrease at 168 h post-induction (682±175 pg^−1^; Fig. 1C, top panel). Intracellular levels of Gag–GFP, as determined by western blotting, showed an increase in expression at early timepoints, reaching a plateau 72 h after induction (Fig. 1C, bottom panel). These results suggest that HIV-1 Gag–GFP is being produced and released at early time points, but later accumulates in THP-1 GagZip macrophages, similar to what is seen in MDMs (Gaudin et al., 2013).

Then, we evaluated by immunofluorescence whether the Gag–GFP clusters observed at 72 h post-induction corresponded to VCCs by evaluating their colocalization with CD81, a tetraspanin described to accumulate at the plasma membrane in T cells and at VCCs in macrophages (Deneka et al., 2007; Jolly and Sattentau, 2007). A significant colocalization of 9.6±1.9% (mean±s.e.m.) was observed between endogenous CD81 and Gag–GFP clusters at 72 h post HIV-1 induction (Fig. 1D,E), suggesting that Gag–GFP clusters in THP-1 GagZip macrophages are VCCs. Given that these compartments could also be formed by the uptake of viral particles from the extracellular medium (Hammonds et al., 2017), we evaluated whether the addition of supernatant from HIV-1-induced macrophages also led to the appearance of VCCs in non-induced cells. Gag–GFP clustering was observed in 21% of non-induced macrophages following 72 h of incubation, representing a smaller amount compared to the 55% observed in cells post-HIV-1 induction (Fig. S1A,B). These results suggest that *de novo* synthesized Gag–GFP leads to VCC formation in THP-1 GagZip macrophages with only a minor contribution from extracellular viral particles, as observed in human macrophages (Hammonds et al., 2017). Endogenous CD81 accumulated at specific sites near the plasma membrane in a time-dependent manner, along with Gag–GFP (Fig. S1C). This pattern resembled the observed colocalization pattern in HIV-1-infected MDMs (Fig. S1D,E), supporting the hypothesis that Gag–GFP clusters in THP-1 GagZip macrophages are VCCs.

Interestingly, we observed that the CD81 immunofluorescence signal near the plasma membrane was stronger in macrophages in which HIV-1 was induced compared to that seen in control conditions (Fig. 1D). Given that CD81 is redistributed and accumulated at HIV-1 assembly sites (Jolly and Sattentau, 2007), we evaluated CD81 levels at the plasma membrane by flow cytometry in THP-1 GagZip macrophages 72 h post HIV-1 induction. Gag–GFP-expressing macrophages showed a significant enrichment of CD81 at the plasma membrane compared to that in cells that do not express Gag–GFP and in non-induced cells. In contrast, we did not observe any change in total levels of CD81 as determined by western blotting (Fig. S1F,G), suggesting that HIV-1 does not increase CD81 expression but repositions it to the plasma membrane, as previously observed in HIV-infected MDMs (Deneka et al., 2007). Additionally, to validate that THP-1 GagZip macrophages form VCCs, we also evaluated cell-to-cell transmission by co-culturing macrophages with SupT1T cells, as VCCs participate in the spreading of HIV-1 by cell-to-cell transmission (Carr et al., 1999; Sharova et al., 2005). At 24 h after co-culturing, we detected the presence of Gag–GFP in 3.8% of T cells, when cell-to-cell contact was allowed. No cell-free transmission was observed when macrophages and T cells were separated by a membrane insert, consistent with this cell model being unable to produce infectious virus particles (Fig. S1H). These results support that our model forms VCCs that are able to transfer Gag from macrophages to T-cells, as described in infected human macrophages (Gousset et al., 2008).

Finally, we evaluated the Gag–GFP clusters observed at 72 h post HIV-1 induction by electron microscopy. Ultrastructural analyses revealed that HIV-1-induced macrophages exhibited vacuolar compartments with electron-dense clusters on their luminal side and some with spherical electron-dense particles with the morphology and approximate size of virus-like particles (Fig. 1F) (Pankrac et al., 2018). Thus, the THP-1 GagZip macrophages we evaluated largely bear the characteristics of VCCs as observed in infected MDMs, including colocalization and clustering of Gag with CD81, concomitant with accumulation of Gag within the cells, and the ability to transfer Gag to T cells through cell-to-cell contact, confirming this cell line as a useful tool to study HIV-1 in the context of VCCs.

### LELs are present at VCCs, as well as HIV-1 Gag at LEL membranes

**Fig. 2.**
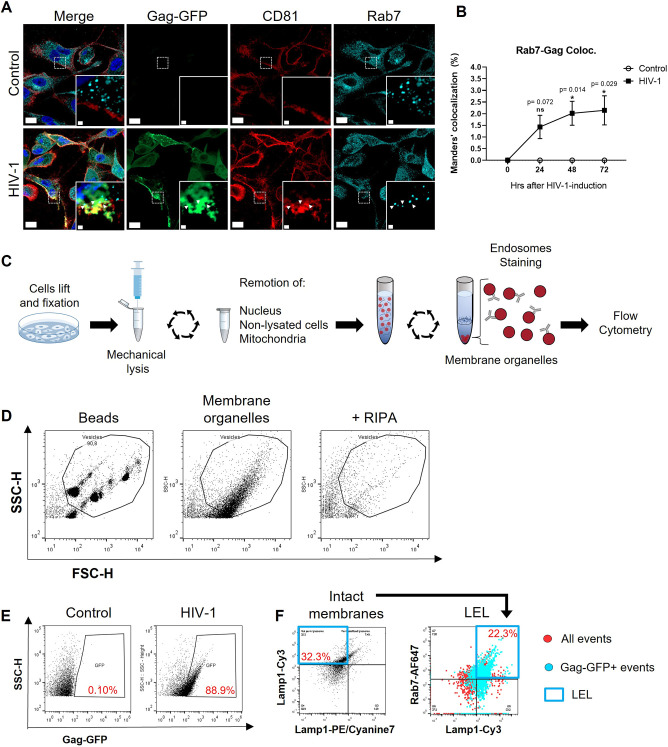
**LELs colocalize with VCCs as well as HIV-1 Gag is present on LEL membranes.** (A) Confocal microscopy immunofluorescence from fixed THP-1 GagZip macrophages evaluated for Gag–GFP expression (green), the VCC marker CD81 (red) and the LEL marker Rab7 (cyan) 72 h after HIV-1 induction or non-induced macrophages (control). White arrowheads indicate triple colocalization between all markers. Scale bars: 15 µm (main image); 1 µm (inset). (B) From A. Bar graph representing Manders' percentage of colocalization Rab7 over Gag–GFP evaluated at 0, 24, 48 and 72 h after HIV-1 induction for control (No Dox) and HIV-1 induced (Dox) THP-1 GagZip macrophages. (C) Scheme depicting the protocol to isolate membrane organelles, stain them, and evaluate the endosomal populations within by flow cytometry. (D) Dot plots depicting control parameters to set up the flow cytometer to detect endosomal organelles. Left plot depicts the beads used to calibrate the equipment by size, gating on events that are between 0.11 to 1.3 µm in size. Middle and right plots represent the events obtained from macrophage lysates in the size range from the left plot, with or without the addition of RIPA to disrupt membrane organelles. (E) Dot plots from THP-1 GagZip macrophage lysates after determining size range for endosomes, showing the detection of Gag–GFP under control or HIV-induced conditions at 72 h post-induction. Percentages indicative of positive population from the total number of detected events. (F) LEL population from the isolated membrane organelles mixture from THP-1 GagZip macrophages 72 h after HIV-induction. Intact membranes were identified by co-staining with antibodies against luminal and cytosolic side of Lamp1 (left panel, blue box. Lamp1-Cy3^+^/Lamp1-PE.Cyanine7^-^). From here, LELs were detected by the co-staining of Rab7 and cytosolic Lamp1 (Lamp1-Cy3^+^/Rab7^-+^). The right panel depicts all events (red dots) detected, highlighting in a blue box LELs. Additionally, cyan circles represent events that are also positive for HIV-1 Gag-GFP. Percentages indicative of positive population from the total number of detected events. All data representative from three independent experiments. Graphs are presented as mean±s.e.m. Statistical analyses in B were performed by two-way ANOVA, using Sidak's post hoc test to compare each time-point between control versus HIV-1 induction conditions. *P<0.05; ns, not significant.

We next evaluated the presence of LELs at VCCs by immunofluorescence at 72 h, as it was the first time point that VCCs were observed post HIV-1 induction. We evaluated endogenous levels of Rab7 [herein referring to Rab7A, a small GTPase commonly used as a LEL marker (Cabukusta and Neefjes, 2018)] along with CD81 and Gag–GFP to detect VCCs. The Manders' overlap coefficient indicated that there was a time-dependent increase in LELs colocalizing with Gag clusters, having a fraction of 2% (which is a significant change) Rab7^+^ colocalization 72 h post HIV-1 induction compared to that in non-induced macrophages (Fig. 2A,B). To further support the hypothesis that LELs are involved in HIV-1 trafficking, we evaluated the presence of Gag–GFP at LEL membranes using a second approach, involving the subcellular fractionation of membrane organelles coupled to flow cytometry (de Araujo et al., 2015; Lamberti et al., 2015). At 72 h post HIV-1 induction, THP-1 GagZip macrophages were subjected to gentle mechanical lysis, and membrane organelles from the cytoplasmic fraction were enriched by ultracentrifugation. LELs were detected by flow cytometry by co-staining cytosolic Lamp1 and Rab7, following a staining protocol for extracellular vesicles (Fig. 2C) (Welsh et al., 2021; Maia et al., 2020). The flow cytometer was calibrated to detect only events that ranged in size from 0.11 to 1.3 µm, as LEL have a size of 0.2–0.6 µm (Barral et al., 2022). Additionally, we evaluated the presence of membranous organelles by adding a strong detergent before acquisition, which dramatically decreased the number of events detected in the expected size range (Fig. 2D).

When evaluating for the presence of Gag–GFP after subcellular fractionation, 88.9±0.3% (mean±s.e.m.) of the detected events were positive for Gag–GFP (Fig. 2E). After identifying LELs within the membrane organelles (cytosolic Lamp1^+^ and Rab7^+^), 20% corresponded to LELs and, to our surprise, 90±0.5% of them were positive for Gag–GFP (Fig. 2F), strongly suggesting an interrelationship between LELs and Gag, with a minor fraction colocalizing at VCCs 72 h post HIV-1 induction (Fig. 2A). These results support the hypothesis that LEL function as a carrier for intracellular HIV-1 Gag translocation within macrophages.

### HIV-1 repositions LELs towards the plasma membrane in macrophages

**Fig. 3.**
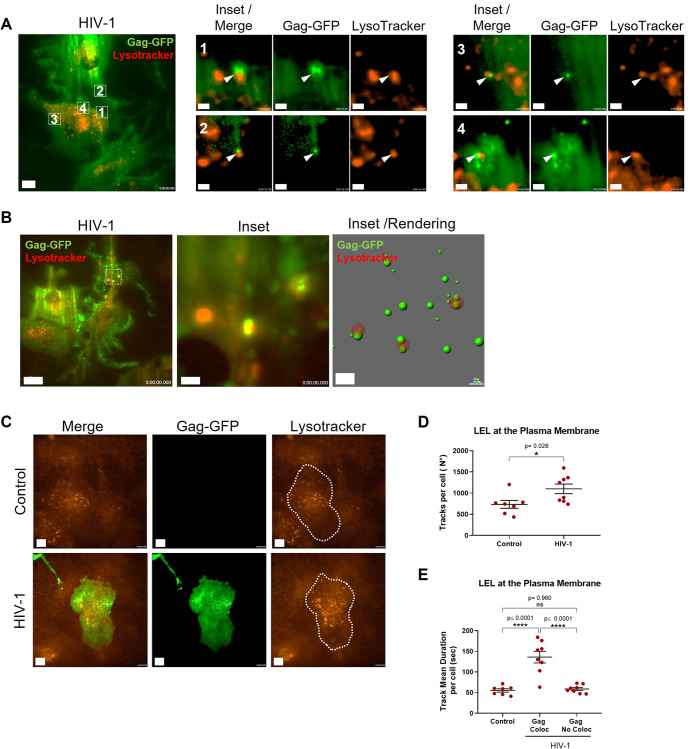
**HIV-1 Gag expression repositions LELs towards the plasma membrane in macrophages.** (A) Total internal reflection fluorescence (TIRF) microscopy from THP-1 GagZip macrophages at 72 h after HIV-1 induction. Visualization of HIV-1 Gag–GFP (green) and LELs (red) stained with 50 nM LysoTracker Red. Four different regions within one cell are magnified on the right side, depicting individual as well as merged signals. White arrowheads indicate colocalizing signals between Gag-GFP and Lysotracker. Scale bars: 10 µm (main image); 1 µm (magnified views). (B) Same conditions as in A. Occasionally, bigger LELs were observed decorated with several Gag–GFP molecules. A magnified area is shown in insets with the respective spots rendition for Gag–GFP (green) and Lysotracker Red (red) signals. Rendition carried out in Imaris v10.0.0. Scale bars: 15 µm (main image); 2 µm (magnified views). (C) Same conditions as in A. Visualization of HIV-1 Gag–GFP (green) and LELs (red) in control condition or after HIV-1 induction. HIV-1 Gag expression increased the number of LELs detected near the plasma membrane by TIRF. Cell perimeters are indicated with a dashed line. Scale bars: 10 µm. (D) From C. Average number of detected LELs per cell near the plasma membrane detected by TIRF microscopy. Data from control and HIV-1-induced THP-1 GagZip macrophages. Each dot represents the mean value obtained per one cell. (E) From C. Average time (in s) that LELs were detected near the plasma membrane, obtained from control and HIV-1-induced THP-1 GagZip macrophages. LELs were sorted based on distance to Gag–GFP molecules, with colocalizing LELs defined as those less than 0.1 µm away from Gag–GFP at any time point. Each dot represents the mean value obtained per one cell. All data representative from three independent experiments. Graphs are presented as mean±s.e.m. Statistical analyses in D were performed with an unpaired two-tailed *t*-test with Welch's correction; in E, one-way ANOVA using Tukey's post hoc test to compare between all data sets was used. *P<0.05; ****P<0.0001; ns, not significant.

VCCs have been described as plasma membrane invaginations that can be enclosed or transiently connected to the extracellular medium (Nkwe et al., 2016; Gaudin et al., 2013; Bennett et al., 2009) and are often found in close proximity to LELs (Deneka et al., 2007; Pelchen-Matthews et al., 2003). Therefore, we evaluated whether HIV-1 modulates LEL movement toward the plasma membrane using total internal reflection fluorescence (TIRF) microscopy in live-cell settings. At 72 h after HIV-1 induction, we stained LELs with LysoTracker Red and observed them in parallel with Gag–GFP. In agreement with our results on fixed samples (Fig. 1D), we found Gag–GFP clusters close to the plasma membrane, suggestive of VCCs, with LELs colocalizing with them (Fig. 3A; Movie 1). Strikingly, we observed LELs close to the plasma membrane that were static (no movement) and decorated with several Gag–GFP molecules (Fig. 3B; Movie 2). We then analyzed LEL positioning at the plasma membrane and their colocalization with Gag–GFP. We rendered Gag and LEL signals as spots and defined them as colocalizing if less than 0.1 µm apart, as described in the Materials and Methods. Our results indicate that HIV-1 induction in THP-1 GagZip macrophages increased the amount of LELs detected close to the plasma membrane by 33% (1099±113 versus 733±93 mean±s.e.m. number of detected events) when compared to LELs in non-induced macrophages (Fig. 3C,D). Furthermore, when determining the average time that LELs spent near the plasma membrane, LELs colocalizing with Gag–GFP exhibited a significant increase in dwell time (136±14 s; mean±s.e.m.), compared to non-colocalizing LELs (59±3.4 s) and LELs from non-induced macrophages (55±4.0 s; Fig. 3D). These observations are in agreement with our previous findings (Cinti et al., 2017), in which we observed that HIV-1 promoted LEL positioning toward the cell periphery. Overall, our data from fixed samples and live-cell imaging support that HIV-1 Gag colocalizes with LELs, which results in their repositioning towards the plasma membrane, where LELs dwell, and are likely to unload Gag for virus assembly at VCCs.

### HIV-1 Gag co-traffics with LELs, altering their motility

**Fig. 4.**
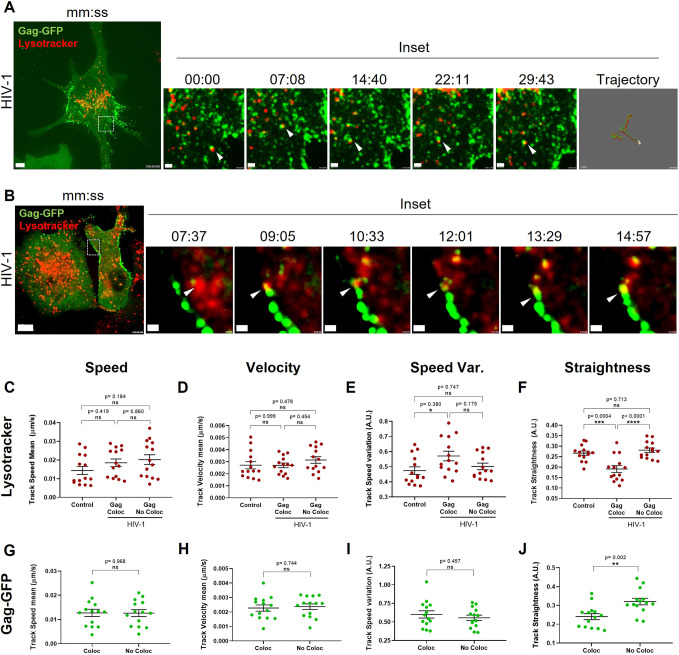
**HIV-1 Gag co-trafficking with LELs modulate their motility.** (A) 2D image from a 3D reconstruction from THP-1 GagZip macrophages 72 h after HIV-1 induction, obtained by live-cell imaging. Visualization of co-trafficking between HIV-1 Gag–GFP (green) and LEL (red) stained with 50 nM LysoTracker Red. A timelapse from the marked area is shown on the right. White arrowheads indicate the co-trafficking between HIV-1 Gag–GFP and a LEL. A rendition of the overall trajectory is depicted at the right corner. Scale bars: 5 µm (main image); 1 µm (magnified views). (B) Same conditions as in A. Visualization of LELs delivering Gag–GFP as a cargo to a previously formed VCC. Timelapse from the marked area is shown on the right. White arrowheads indicate the LELs loaded with Gag–GFP molecules being delivered. Scale bars: 10 µm (main image); and 1 µm (magnified views). (C–F). Single-molecule track analysis. LEL spots were rendered in Imaris, and their motility was analyzed 72 h after control conditions or HIV-1 induction. LELs were analyzed to determine their mean track speed (C), velocity (D), speed variation (E) and straightness (F). LELs were sorted based on their distance to Gag–GFP molecules, with colocalization with LELs (Gag Coloc.) being defined as those that were less than 0.1 µm away from each other at any time point. Each dot represents the mean value obtained per one cell. (G–J) Same tracking analyses as in C–F but performed on Gag–GFP molecules. Gag spots were sorted as colocalizing or not with LELs following the same criteria. Each dot represents the mean value obtained per one cell. All data representative from four independent experiments. Graphs are presented as mean±s.e.m. Statistical analyses in C–F are one-way ANOVA and Tukey's post hoc test to compare between all data sets. For G–J, unpaired two-tailed *t*-test with Welch's correction was used. *P<0.05; **P<0.01; ***P<0.001; ****P<0.0001; ns, not significant. A.U., arbitrary units.

Based on our findings that HIV-1 increases LEL number and the time spent close to the plasma membrane in macrophages, we decided to evaluate the effect of HIV-1 on the entire LEL population. We stained LELs with LysoTracker Red and recorded THP-1 GagZip macrophages, at 72 h post HIV-1 induction, in several optical fields on the *Z*-axis to generate three-dimensional renditions. With this approach, we observed long-lasting interactions between LELs and Gag–GFP, with some co-trafficking for up to 30 min (Fig. 4A; Movie 3). Surprisingly, we observed a population of LELs decorated with Gag–GFP that was unloaded to pre-existing existing Gag clusters (Fig. 4B; Movie 4). We then analyzed LEL and Gag–GFP motility by calculating the mean value per cell for the velocity, speed, speed variation and straightness of their tracks. Overall, we did not observe any significant differences in LEL motility between control and HIV-induced macrophages (Fig. S2A–D). However, after sorting LEL tracks based on whether they colocalized with Gag–GFP or not, we observed that colocalizing Gag-loaded LELs exhibited a significant increase (by 20%) in their speed variation and a decrease (by 28%) in their track straightness, when compared to non-colocalizing LELs and control conditions. These results indicate that Gag-loaded LELs have larger fluctuations in their overall speed, as well as a more stochastic movement compared to Gag-free LELs (Fig. 4C–F). We also determined the abundance of LELs detected under control and HIV-1 induced conditions, but no significant difference was observed (Fig. S2E), indicating that HIV-1 Gag only affects LEL motility and not their biogenesis.

Lysosomes are often associated with protein degradation mediated by the pH-dependent hydrolases in their luminal side (Trivedi et al., 2020). To rule out the possibility that colocalization of Gag–GFP with LELs is related to degradation, we used the same approach, but stained lysosomes with Magic Red, a reagent that emits fluorescence only when degraded by cathepsin B, a lysosome-specific protease (Mort, 2013). We observed that HIV-1 induction increased cathepsin B activity by 10% compared to control macrophages (Fig. S2F). When quantifying the percentage of LELs that colocalized with Gag–GFP, there was no significant difference between results found using Lysotracker Red and Magic Red dyes (Fig. S2G). Both dyes indicated that 6% of LELs colocalized with Gag, correlating with the observed 2% of Rab7^+^ LEL colocalization with Gag at VCCs, observed in fixed samples (Fig. 2B). These results, along with the flow cytometry indicating that Gag is present in more than 90% of LELs (Fig. 2F), mean it is plausible to suggest that the increase in cathepsin B activity is related to excess Gag on LELs. These results could be explained by Gag–GFP being targeted for assembly trafficking on the cytosolic side of LELs instead of their luminal side, where degradative activity takes place (Mort, 2013).

Additionally, we also evaluated Gag particle tracks by sorting them based on their colocalization with LELs, following the same colocalization parameters (less than 0.1 µm apart) used above. Gag–GFP particles did not show any differences in speed, velocity, or speed variation. However, colocalizing Gag-loaded LELs had a significant decrease of 25% in their track straightness (Fig. 4G–J), correlating with the decrease in the track straightness observed in LEL analyses (Fig. 4F). Overall, we demonstrated by live-cell imaging that HIV-1 Gag traffics on LEL cytosolic side to reach VCCs, and their interaction results in altered LEL motility as well as their repositioning towards the plasma membrane.

### Arl8b-mediated LEL repositioning toward the plasma membrane decreases VCCs formation

**Fig. 5.**
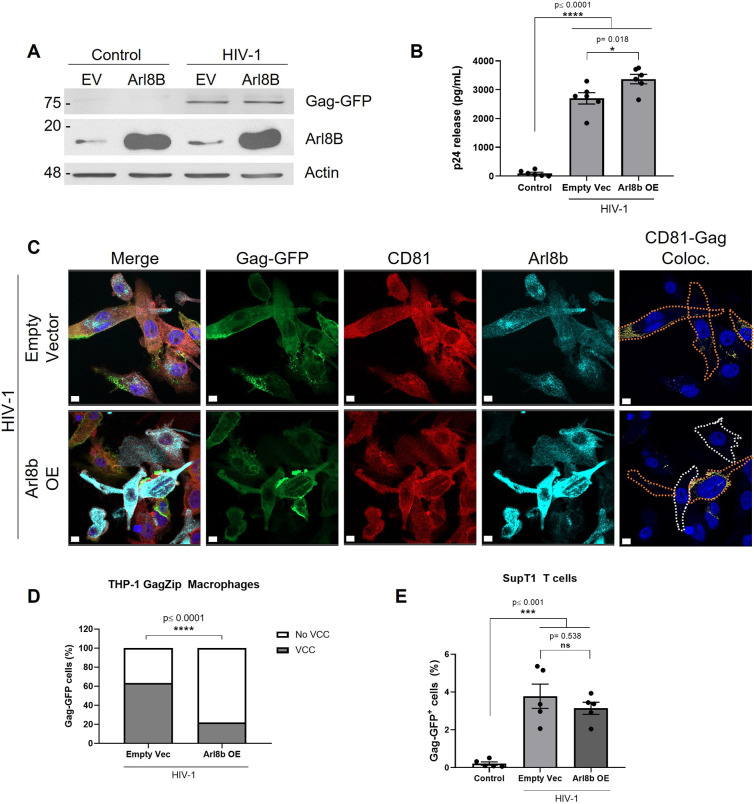
**Arl8b overexpression decreases VCCs formation.** (A) Western blot after simultaneous HIV-1 induction (or control conditions) and transduction with the Arl8b-coding or empty vector. At 72 h after co-treatments, cells were lysed and evaluated for HIV-1 Gag–GFP and Arl8b expression, using β-actin as a loading control. (B) HIV-1 p24 release into the supernatant measured by ELISA. THP-1 GagZip macrophage supernatants were collected 72 h after simultaneous HIV-1 induction (or control conditions) and transduction with the Arl8b-coding or empty vector. (C) Confocal microscopy from fixed THP-1 GagZip macrophages evaluated for Gag–GFP expression (green), the VCC marker CD81 (red) and Arl8b expression (cyan) by immunofluorescence at 72 h after simultaneous HIV-1 induction (or control conditions) and transduction with the Arl8b-coding or empty vector. Cells with VCCs, identified as having colocalizing signal between Gag–GFP and CD81 (yellow signal), are represented on the right-end panel. Cell perimeters are delineated with orange if cells have VCCs, and white otherwise. Scale bars: 10 µm. (D) From C. Percentage of macrophages showing the VCC phenotype, defined by the colocalization between Gag–GFP and CD81. Gag–GFP-expressing cells were counted based on Arl8b overexpression or transduction with an empty vector. (E) Percentage of HIV-1 Gag-GFP+ Sup T1T cells recovered 24 h after co-culture with THP-1 GagZip macrophages after control or HIV-1-induced conditions, transduced with the Arl8b-coding or empty vector as described for C. Cells were analyzed by flow cytometry, detecting Gag–GFP on viable cells only. All data representative from three independent experiments. Graphs are presented as mean±s.e.m. Statistical analyses in B and E were one-way ANOVA, using Tukey's post hoc test to compare between all data sets; for D, an unpaired two-tailed *t*-test with Welch's correction was used comparing between datasets of cells with VCCs. *P<0.05; ***P<0.001; ****P<0.0001; ns, not significant.

Endosomal and lysosomal positioning within the cell is mediated by motor complexes that bind to the cytosolic side of endosomal compartments (Naslavsky and Caplan, 2018). Given the evidence that HIV-1 repositions LELs towards the plasma membrane (Fig. 3), we wondered whether Arl8b, a small GTPase that repositions LELs to the plasma membrane (Jongsma et al., 2020; Khatter et al., 2015), is involved in VCC formation. We evaluated VCC formation in the context of Arl8b overexpression by transduction and after depletion by siRNA transfection, modulating its expression in THP-1 GagZip macrophages in parallel to HIV-1 induction during 72 h. Arl8b overexpression did not affect intracellular levels of HIV-1 Gag as measured by western blotting (Fig. 5A) but increased p24 release into the supernatant by 25% compared to macrophages transduced with an empty vector (2701 ± 198 pg ml^−1^ versus 3369±165 pg ml^−1^, mean±s.e.m.; Fig. 5B). Immunofluorescence analysis revealed that most cells overexpressing Arl8b did not exhibit colocalization between Gag–GFP and CD81 (Fig. 5C), reducing the number of cells displaying VCCs by 41% (Fig. 5D). By contrast, when LELs were repositioned to the juxtanuclear region by Arl8b depletion (Khatter et al., 2015), there were no effects on HIV-1 Gag intracellular levels, on p24 release or on VCC formation (Fig. S3A–D).

Based on our observation that Arl8b overexpression decreased VCC formation, coupled with the role of VCCs in HIV-1 cell-to-cell transmission (Lopez et al., 2019), we evaluated whether Arl8b overexpression would affect macrophage-to-T cell transmission. As mentioned above, we co-cultured THP-1 GagZip macrophages with SupT1 CD4+ T cells for 24 h, and then measured HIV-1 transmission by evaluating the presence of Gag–GFP in T cells by flow cytometry. Arl8b overexpression did not affect HIV-1 cell-to-cell transmission to T cells when compared to macrophages transduced with an empty vector (Fig. 5E). Notably, Arl8b overexpression did not affect the percentage of macrophages expressing HIV-1 Gag–GFP (Fig. S3E), ruling out the possibility that the decrease in VCC formation is explained by a lower number of HIV-1-expressing macrophages. Overall, Arl8b overexpression negatively affects VCC formation, increasing the release of viral proteins, whereas its depletion does not have any effect on VCC formation. The small GTPase Rab7 is known to be displaced from LELs in the presence of Arl8b (Jongsma et al., 2020). Based on our results, HIV-1 likely hijacks Rab7-interacting motor proteins to direct LELs towards VCCs, a process antagonized by Arl8b overexpression. HIV-1 induction in THP-1 GagZip macrophages did not alter the expression of Rab7 nor Arl8b (Fig. S3F).

### Inhibition of N-myristoylation by PCLX-001 blocks VCCs formation

**Fig. 6.**
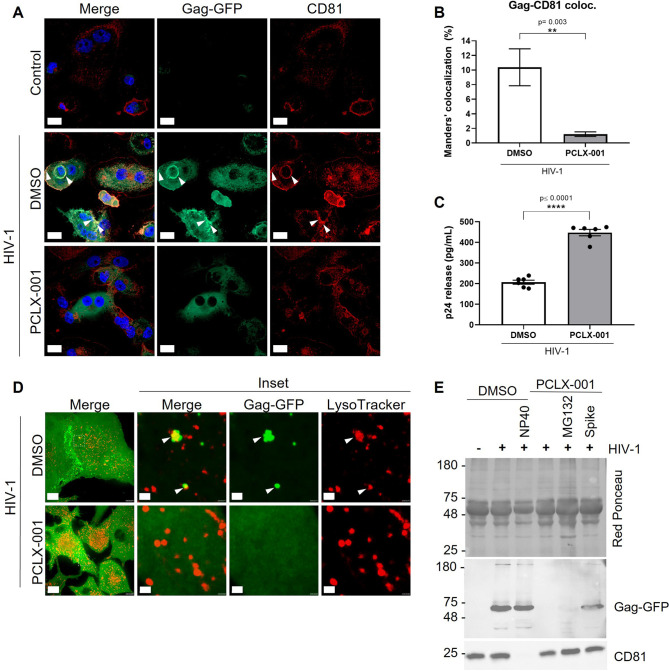
**Inhibition of N-myristoyltransferases by PCLX-001 impedes VCCs formation and HIV-1 assembly in macrophages.** (A) Confocal microscopy from fixed THP-1 GagZip macrophages evaluated by immunofluorescence for Gag–GFP expression (green) and the VCC marker CD81 (red). Samples were collected 72 h after simultaneous HIV-1 induction (or control conditions) and treatment with 1 µM PCLX-001 or DMSO. Scale bars: 20 µm. (B) From A. Bar graph representing Manders' percentage of colocalization of Gag–GFP over CD81, evaluated 72 h after HIV-1 induction alongside DMSO or 1 µM PCLX-001 treatment. (C) HIV-1 p24 release into the supernatant measured by ELISA. THP-1 GagZip macrophage supernatants were collected 72 h after simultaneous HIV-1 induction, and DMSO or 1 µM PCLX-001 treatment. (D) 2D image from a 3D reconstruction of THP-1 GagZip macrophages 72 h after HIV-1 induction and treatment with 1 µM PCLX-001 or DMSO treatment, obtained by live-cell imaging. Macrophages were observed for Gag–GFP signal (green) and LELs (red) stained with 50 nM LysoTracker Red. White arrows indicate co-trafficking between Gag–GFP and LELs. Scale bars: 10 µm (main image); 1 µm (magnified views). (E) Western blot to evaluate viral assembly by using concentrated viral particles from the supernatant 72 h after HIV-1 induction in THP-1 GagZip Macrophages, treated with DMSO or 1 µM PCLX-001. Red Ponceau staining was used as a loading control, and membrane was incubated with anti-HIV-1 p24 or anti-CD81. All data representative from three independent experiments. Graphs are presented as mean±s.e.m. Statistical analyses in B and C were obtained with unpaired two-tailed *t*-test with Welch's correction. **P<0.01; ****P<0.0001.

To assemble new virus particles, HIV-1 requires Gag to multimerize and bind to membranes (Freed, 2015). The latter step is mediated by the matrix domain within Gag, which recognizes the phosphatidylinositol 4,5-bisphosphate [phospholipid PI(4,5)P2] through the myristic acid located at the N-terminal region of Gag (Ono et al., 2004; Favard et al., 2019; Linder, 2009; Zhou et al., 1994). PI(4,5)P2 is mainly present at the plasma membrane, but is also found at LELs (Wallroth and Haucke, 2018; Wills and Hammond, 2022). Based on our finding that Gag co-traffics with LELs to reach VCCs, we decided to evaluate whether the ability of the HIV-1 Gag protein to bind to membranes would affect VCCs formation. To this end, THP-1 GagZip macrophages were incubated with PCLX-001, a clinically validated first-in-class small-molecule inhibitor of NMT1/2, resulting in the abrogation of N-myristoylation on newly synthesized proteins (Sangha et al., 2022; Beauchamp et al., 2020; Sangha et al., 2024). We evaluated the reduction in the integration of myristic acid by adding an alkynyl-myristate analog that reacts with an azido-fluorophore and emits a detectable signal. THP-1 GagZip macrophages were incubated with increasing concentrations of PCLX-001 (from 0.1 to 2 µM) and the myristic acid signal was evaluated by confocal microscopy. We observed that even the lowest concentration of PCLX-001 significantly decreased myristic acid integration, resulting in a 38% reduction in the mean fluorescence intensity (MFI) compared to the DMSO vehicle control (16,691±638 MFI versus 10,315±860 MFI, respectively; mean±s.e.m.). Higher concentrations of PCLX-001 decreased myristic acid integration by almost 60% (i.e. 2 µM, 7834±507 MFI), indicating a significant inhibition of the N-myristoylation process (Fig. S4A,B). Additionally, only treatment with 2 µM PCLX-001 resulted in significant levels of toxicity, whereas lower concentrations did not differ in cell viability from THP-1 GagZip macrophages treated with DMSO vehicle (Fig. S4C). To evaluate this effect in the context of VCCs, HIV-1 expression was induced concurrently with the addition of 1 µM PCLX-001 for 72 h. Strikingly, PCLX-001 completely inhibited VCC formation, as Gag–GFP did not cluster or colocalize with CD81 as observed in DMSO conditions (Fig. 6A,B). Intriguingly, when we measured HIV-1 p24 release, we observed a significant dose-dependent increase in p24 release compared to that in the DMSO vehicle condition (207±10 pg/ml versus 599±58 pg/ml, mean±s.e.m.; Fig. 6C; Fig. S4C). Additionally, we evaluated the effect of PCLX-001 in the co-trafficking of Gag–GFP with LELs in live cells. 1 µM PCLX-001 inhibited Gag–GFP co-trafficking with LELs (Fig. 6D), indicating that N-myristoylation plays a key role in Gag binding to LEL membranes and the formation of VCCs. The observation that the lack of myristoylation increased viral release was unexpected, as inhibition of HIV-1 membrane binding impairs virus particle formation (Zhou et al., 1994). We then evaluated whether HIV-1 was indeed being assembled, as p24 quantification by ELISA does not distinguish between p24 as part of virus particles or as secreted protein (Geraerts et al., 2006). To address this question, we concentrated the virus particles in the supernatant by ultracentrifugation on top of a sucrose cushion, to remove soluble proteins and cell debris (Waheed et al., 2009). Confirming our hypothesis, we did not detect HIV-1 particles by western blotting when cells were incubated with 1 µM PCLX-001, but the phenotype was recovered when PCLX-001-treated macrophages were spiked with supernatant from HIV-1 induced DMSO-treated macrophages (Fig. 6E). We incubated macrophages with 5 µM MG132, a proteasomal-mediated degradation inhibitor, to evaluate whether PCLX-001 prevents viral release by inducing degradation of Gag. We did not observe a recovery in virus particle release after MG132 treatment, suggesting that the inhibition of N-myristoylation by PCLX-001 impedes virus assembly and induces the secretion of soluble Gag into the supernatant (Fig. 6E). This phenotype was also observed in HIV-1 transfected HeLa cells (Fig. S4E), suggesting that N-myristoylation is key for virus assembly at VCCs and at the plasma membrane. Importantly, PCLX-001 did not affect HIV-1 Gag expression, nor did HIV-1 modulate NMT1 or NMT2 expression levels in THP-1 GagZip macrophages (Fig. S4F).

### PCLX-001 treatment decreases the presence of Gag–GFP in endosomal populations

**Fig. 7.**
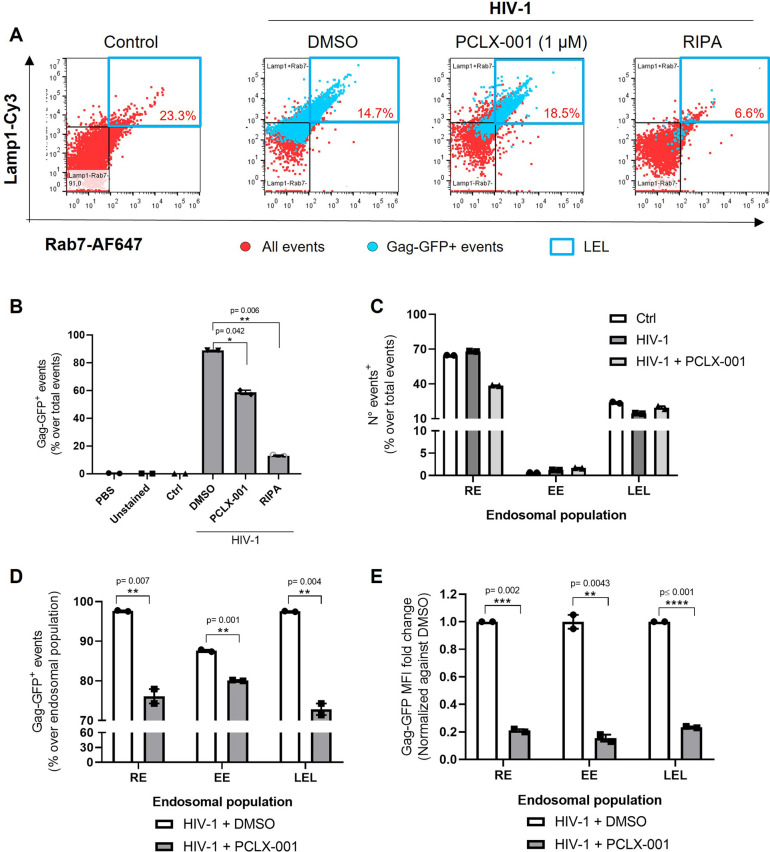
**Treatment with PCLX-001 decreases HIV-1 Gag on endosomal populations.** (A) Dot plots from THP-1 GagZip macrophages after enrichment of endosomes 72 h after simultaneous HIV-1 induction (or control conditions) and treatment with DMSO, 1 µM PCLX-001 or RIPA buffer, analyzed by flow cytometry. Plots depict the LEL population defined by co-staining of Rab7 and Lamp1 membrane organelles isolated (highlighted in a blue box). Red dots represent all events, whereas cyan dots represent events that are positive for HIV-1 Gag–GFP signal. Percentages in red indicative of positive LEL population from the total number of detected events. (B) Bar graphs representing the percentage of Gag–GFP^+^ events after membrane organelle fractionation obtained from PBS only, the unstained fraction, control conditions or HIV-1-induced samples treated with DMSO, 1 µM PCLX-001 or RIPA buffer. (C) Bar graphs representing the total amount of events, indicating the percentage of events from non-induced samples (control), or HIV-1 induced and treated with DMSO or 1 µM PCLX-001. Events were sorted by endosomal populations, identifying recycling endosomes (RE. CD71-BrilliantViolet 421^+^), early endosomes (EE. EEA1-mFluorViolet 610^+^) and LELs (Lamp1-Cy3^+^/Rab7-AF 647^+^). (D) From C. Percentage of Gag–GFP^+^ events within different endosomal populations, normalized against the total amount of events within each endosomal population under DMSO conditions. (E) From C. Gag–GFP MFI fold change between different endosomal populations, normalized against their respective MFI from each population under DMSO conditions. All data representative from two independent experiments. Graphs are presented as mean±s.e.m. Statistical analysis in B is one-way ANOVA, using Dunnett's post hoc test to compare each data set against HIV-1 induced sample treated with DMSO. In D and E, an unpaired two-tailed *t*-test within each endosomal population was perfomed. **P<0.01; ***P<0.001; ****P<0.0001.

To further examine the effect of PCLX-001 on HIV-1 interaction with LELs, we performed the endosomal flow cytometry approach outlined previously (Fig. 2C), but evaluated LELs along with recycling and early endosomes, using antibodies targeting CD71 (or transferrin receptor protein 1) and EEA1, respectively (Elkin et al., 2016; Kobayashi and Fukuda, 2013). The addition of 1 µM PCLX-001 decreased the presence of Gag–GFP by 31% in the LEL population compared to its counterpart in DMSO vehicle conditions (90%±0.5 versus 59%±0.9, mean±s.e.m.; Fig. 7A). Furthermore, 1 µM PCLX-001 reduced by 30% the number of Gag–GFP^+^ events after membrane organelle fractionation compared to the number seen in the DMSO vehicle condition, indicating that PCLX-001 dramatically diminishes HIV-1 Gag anchoring to endosomal membranes in general (Fig. 7B). Importantly, when we sorted membrane organelles by endosomal subpopulations, most of the detected endosomes corresponded to recycling endosomes, followed by LELs and early endosomes (Fig. 7C). Notably, HIV-1 induction did not affect the overall number of detected endosomes per subpopulation, whereas PCLX-001 affected only the amount of detected recycling endosomes. We then determined the percentage of Gag–GFP^+^ events within specific endosomal populations as well as their respective MFI. We observed that, similar to our previous observation in LELs (Fig. 2F), Gag–GFP is present in the majority of detected recycling and early endosomes. The addition of 1 µM PCLX-001 significantly decreased the percentage of Gag–GFP^+^ events detected in each endosomal population (Fig. 7D). More strikingly, PCLX-001 significantly reduced the Gag–GFP MFI in all populations (by 80%) when compared to that for the DMSO vehicle condition. These results suggest that NMT1/2 inhibition decreases the overall presence of HIV-1 Gag in endosomal membranes, highlighting the relevance of endosomal compartments during HIV-1 infection, as Gag is highly enriched in all of them. Thus, Gag N-myristoylation might represent a candidate target in future therapeutics.

### PCLX-001 treatment inhibits VCCs formation in human macrophages

**Fig. 8.**
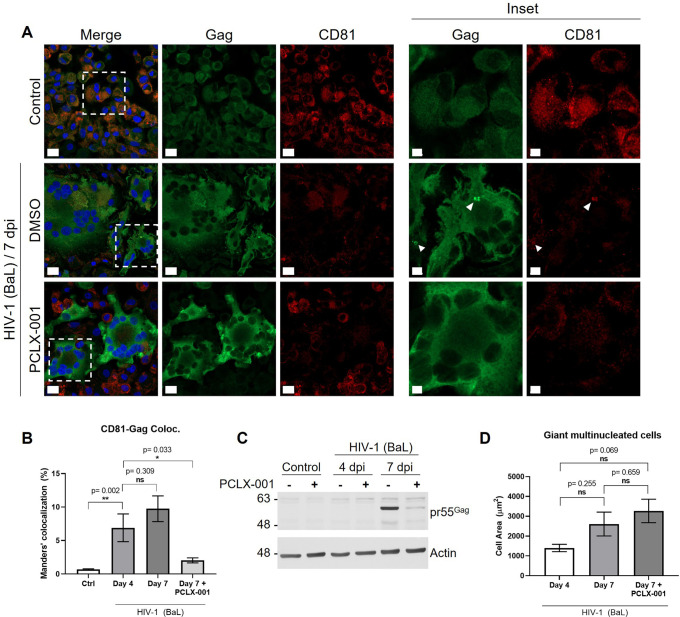
**Treatment with PCLX-001 inhibits VCC formation in human macrophages.** (A) Confocal microscopy from fixed human uninfected MDMs (control) or MDMs infected with HIV-1 BaL for 4 or 7 days, with or without 72 h treatment of PCLX-001 (1 µM). Samples were stained by immunofluorescence to detect Gag (green) and CD81 (red). Right side panels show magnified areas (insets), where white arrowheads indicate colocalization between Gag and CD81 clusters. Scale bars: 15 µm (main images); 5 µm (magnified views). (B) From A. Bar graph representing Manders' percentage of colocalization of CD81 over Gag–GFP, evaluated at 7 days after HIV-1 BaL infection, with or without 72 h treatment of PCLX-001 (1 µM). Uninfected condition labeled as Ctrl. (C) Western blot from human uninfected MDMs (control) or MDMs infected with HIV-1 BaL for 4 or 7 days, with or without 72 h treatment of PCLX-001 (1 µM). The membrane was evaluated for HIV-1 Gag, using β-actin as a loading control. (D) Bar graphs representing the mean cell area of giant multinucleated cells after HIV-1 BaL infection, during 4 or 7 days, with or without 72 h treatment of PCLX-001 (1 µM). All data representative from two independent experiments. Graphs are presented as mean±s.e.m. Statistical analysis in B and D is one-way ANOVA, using Dunnett's post hoc test to compare each data set against HIV-1 4 days after infection B, or using Tukey's post hoc test to compare between all data sets D. *P<0.05; **P<0.01; ns, not significant.

To evaluate the efficacy of PCLX-001 in the context of a fully infectious virus, we infected MDMs with the HIV-1 BaL strain for 4 days and then treated them with 1 µM PCLX-001 for an additional 72 h. As observed in THP-1 GagZip macrophages, 1 µM PCLX-001 significantly decreased VCC formation, Gag clustering and Gag colocalization with CD81 (Fig. 8A). Interestingly, PCLX-001 also decreased intracellular levels of HIV-1 Gag compared to that seen in non-treated MDMs 7 days after infection (Fig. 8B). HIV-1 infection in macrophages results in the fusion of cells, forming giant multinucleated cells (Han et al., 2022; Mascarau et al., 2023). The addition of 1 µM PCLX-001 did not affect this phenotype in HIV-1 BaL-infected MDMs (Fig. 8D). PCLX-001 proved to be an efficient tool to block the formation of VCCs and, thus, the intracellular accumulation of HIV-1 particles in human macrophages, placing us one step closer to counteract the HIV-1 reservoir stablished in infected people.

## DISCUSSION

Macrophages represent an important viral reservoir. Although initially controversial, macrophages have been suggested as one of the main HIV-1 targets from where viral rebound occurs in the absence of cART. A humanized myeloid-only mouse model, depleted of T cells, has sustained HIV-1 replication (Honeycutt et al., 2017, 2016). Similarly, in a rhesus macaque model infected with simian immunodeficiency virus (SIV), SIV can rebound from infected macrophages following cART interruption, making macrophages an important cell type to consider in future curative strategies (Avalos et al., 2017; Micci et al., 2014). However, a complete picture of how HIV-1 orchestrates assembly, especially at VCCs in infected macrophages, remains to be established. Most research has been conducted on HeLa cells or human CD4+ T cells, in which virus assembly occurs at the plasma membrane (Joshi et al., 2009). HIV-1 infection in macrophages is not always successful because of several antiviral factors (Cassol et al., 2006; Swingler et al., 2007; Yuan et al., 2017), resulting in a minor proportion of cells forming VCCs. In this report, we show that macrophages derived from the promonocytic cell line THP-1 GagZip readily form VCCs as seen in HIV-1-infected MDMs (Tan and Sattentau, 2013; Joshi et al., 2009). Therefore, the THP-1 GagZip model is a valuable tool for studying late stages of HIV-1 infection in macrophages. Using this model, we identified an interrelationship between HIV-1 Gag and LELs, demonstrating that LELs play an important role in HIV-1 Gag trafficking to VCCs. Finally, blocking N-myristoylation completely abrogated HIV-1 assembly and VCC formation in macrophages, which could lead to the development of novel therapies to target this viral reservoir.

We observed that Gag–GFP accumulated at specific locations near the plasma membrane 72 h after virus induction, colocalizing with CD81 (Fig. 1). Our results are in agreement with earlier findings in MDMs, in which HIV-1 was shown to accumulate at VCCs in a time-dependent manner (Gaudin et al., 2013) along with the tetraspanins CD81, CD9 and CD53 (Deneka et al., 2007). Although THP-1 GagZip VCCs were not overfilled with viral particles, as described in HIV-1-infected MDMs using electron microscopy (Nkwe et al., 2016; Gaudin et al., 2013; Bennett et al., 2009), we provide robust evidence to support that HIV-1 induction resembles several aspects of HIV-1 infection in MDMs, including CD81 redistribution to the plasma membrane, intracellular virus accumulation and transmission from macrophages to CD4+ T cells (Fig. S1). Previous research using MDMs showed that, following the required 7 days to differentiate macrophages, HIV-1 Gag–GFP accumulation is apparent at 6–7 days post-infection (Alteri et al., 2022; Graziano et al., 2022; Gaudin et al., 2013). THP-1 GagZip monocytes require 4 days to differentiate into macrophages and 3 days of HIV-1 induction to observe VCCs, reducing the required time by half compared to human MDMs. In addition, the THP-1 GagZip cell line does not produce infectious particles, providing a tool to study the late stages of HIV-1 infection under lower-level biosafety conditions.

Here, for the first time, co-trafficking of HIV-1 Gag with LELs was observed in live macrophages. Gag colocalization altered LEL motility, as well as repositioning them towards the plasma membrane. Our earlier work also showed the colocalization of HIV-1 Gag with LELs under various conditions (Lehmann et al., 2009). In addition, we have previously shown that HIV-1 counteracts the repositioning of LELs mediated by different stressors involving mTORC1 activation (Cinti et al., 2017), supporting the notion that LELs play an important role during the HIV-1 infectious cycle.

LELs are usually associated with protein degradation or recycling, but they are highly dynamic signaling hubs that extensively communicate with other cellular structures through membrane contact sites (Ballabio and Bonifacino, 2020). For example, mTORC1, a master regulator of cellular biosynthetic pathways, is activated on the LEL surface (Sancak et al., 2010); LELs transport RNA granules by anchoring them through annexin A11 (Liao et al., 2019), and can sustain localized translation by transporting ribosomes to the mitochondria (Cioni et al., 2019). In macrophages, VCCs are surrounded by focal adhesion-like structures (Rodrigues et al., 2022). Focal adhesions are associated with mRNA translation, as they colocalize with active mTORC1 on lysosomes (Rabanal-Ruiz et al., 2021). Therefore, HIV-1 Gag trafficking on LELs toward VCCs might play a role in coordinating HIV-1 gene expression and assembly. Here, we demonstrated that HIV-1 increased the speed variation of LELs while decreasing the straightness of their trajectory (Fig. 4). This could be explained by the increased speed of LELs as they transit toward VCCs, followed by its decrease at the time of close contact, presumably when unloading Gag on VCCs. Importantly, Lysotracker Red did not stain Gag–GFP clusters during live-cell imaging, indicating that the VCC pH is not acidic, supporting earlier work demonstrating that VCCs do not fuse with LELs (Jouve et al., 2007; Welsch et al., 2007).

Given that the small GTPase Arl8b is associated with LEL positioning to the cell periphery (Jongsma et al., 2020), we queried whether HIV-1-mediated repositioning of LELs could involve Arl8b. Unexpectedly, Arl8b overexpression decreased the number of macrophages that exhibited VCCs (Fig. 5). Earlier studies on small GTPases in the context of HIV-1 concluded that Rab7 plays an important role in viral assembly (Caillet et al., 2011). Additionally, Arl8b bound to LELs displaces Rab7 from them (Jongsma et al., 2020). Considering that, and that Arl8b overexpression would decrease the availability of membrane-bound Rab7, our findings suggest that HIV-1 Gag probably directs LEL movement towards VCCs by interacting with a Rab7-related motor complex, although additional evidence is required.

HIV-1 assembly requires Gag multimerization and binding to membranes (Freed, 2015; Janvier et al., 2011). The latter step is mediated by the matrix domain within Gag, which recognizes PI(4,5)P2 via the myristic acid found at N-terminus of Gag (Zhou et al., 1994; Ono et al., 2004; Favard et al., 2019; Linder, 2009). Myristoylation plays multiple roles in the regulation of membrane structure, signaling and transport (Wallroth and Haucke, 2018; Wills and Hammond, 2022), and is essential for HIV-1 replication (Hermida-matsumoto and Resh, 2000; Peng and Robert-Guroff, 2001) as well as replication for other viruses (Cao et al., 2020; Priyamvada et al., 2022). PI(4,5)P2 is mainly present at the plasma membrane but is also present in endosomal and lysosomal membranes (Wallroth and Haucke, 2018; Wills and Hammond, 2022). Considering the high abundance of HIV-1 Gag on endosomes (Fig. 7), and only the small percentage colocalizing with VCCs (Figs 2 and 4), it would be interesting to evaluate whether the presence of PI(4,5)P2 on LELs is related to HIV-1 Gag co-trafficking towards VCCs.

By inhibiting NMT1/2 activity in macrophages using PCLX-001, we blocked the co-trafficking of HIV-1 Gag with LELs, impeding virus assembly and VCCs formation in our model (Fig. 6) and in infected human MDMs (Fig. 8). These observations agree with previous research in which mutation of the myristoylation sequence in HIV-1 Gag abrogated the colocalization with LELs (Lehmann et al., 2009). NMT1 is also associated with the activation of mTORC1 on lysosomes (Chen et al., 2020), which is also modulated during HIV-1 replication (Cinti et al., 2017), and the lack of N-myristoylation of Gag impairs its binding to PI(4,5)P2.

We observed that HIV-1 Gag is present in all endosomal populations, being highly enriched in LELs. This observation agrees with previous research on HT-1080 human epithelial cells, where the trafficking of HIV-1 *gag* mRNA within different endosomal populations was evaluated (Molle et al., 2009), which observed that *gag* mRNA is present in all endosomal compartments, but mainly in LELs. Here, we observed that PCLX-001 decreased the presence of HIV-1 Gag in all evaluated endosomal subpopulations (Fig. 7). Our results highlight the importance of N-myristoylation in HIV-1 targeting endosomes and suggest that the inhibition of N-myristoylation abolishes HIV-1 assembly, as we were not able to recover assembled particles in cells treated with PCLX-001 (Fig. 6). Given that PCLX-001 inhibits the overall N-myristoylation in cells, the effects observed on HIV-1 trafficking and assembly in macrophages could be related to a dependence of this modification on other host or viral proteins. Importantly, PCLX-001 inhibited VCC formation in MDMs, raising the possibility of repurposing this drug to target HIV-1 assembly in macrophages.

In conclusion, using the THP-1 GagZip macrophage model, we have demonstrated that HIV-1 Gag interacts with and hijacks LELs to enable recruitment to VCCs, altering LEL positioning and motility. We also demonstrated that the lack of this interaction, upon blocking N-myristoylation using PCLX-001, results in a complete inhibition of HIV-1 assembly, moving us one step closer to a complete understanding of HIV-1 trafficking in infected macrophages and possible targets for future treatments.

## MATERIALS AND METHODS

### Reagents and antibodies

All reagents and antibodies used in the experimental procedures are detailed in Table S1.

### Cell culture and THP-1 GagZip generation

THP-1 GagZip monocytes and Sup-T1 T cells (ATCC CRL-1942) were cultured in RPMI supplemented with 10% FBS, 100 U of penicillin, 0.1 mg/ml streptomycin and 0.05 mM 2-mercaptoethanol. HeLa (ATCC CCL-2) and HEK-293T cells (ATCC CRL-11268) were cultured in DMEM supplemented as above minus 2-mercaptoethanol, defined as complete medium hereafter. All cells were incubated at 37°C and 5% CO_2_. Washing steps between different protocols were carried out with PBS without Ca^2+^ and Mg^2+^ (D-PBS).

To generate the HIV-inducible THP-1 GagZip cell line, THP-1 cells (ATCC TIB-202) were transduced with a derivative of a previously described Dox-regulated HIV-1 provirus in which the Nef protein has been replaced by the reverse tetracycline-controlled transcriptional activator gene (rtTA) (Das et al., 2004; Zhou et al., 2006). The construct was generated by replacing the fragment between Spe1/Mls1 from the HIV-1 rtTA Δmls plasmid with the same fragment from the pNL4-3 GagzipGFP vector, where Gag is fused in frame to GFP at its c-terminus. The nucleocapsid (NC) portion of Gag was replaced by a leucine zipper sequence (zip) of the human cAMP response element-binding protein (CREB) protein, which allows Gag multimerization while decreasing vRNA binding (Accola et al., 2000; Mattei et al., 2015). The HIV-1 Pol segment has mutations in the protease (PR) and reverse transcriptase (RT) domains, resulting in replication-incompetent HIV-1 particles. After transduction, clonal cell lines were sorted by FACS based on THP-1 GagZip monocytes GFP expression after incubation with 2 µg/ml of doxycycline.

### THP-1 GagZip macrophage differentiation and HIV-1 induction

THP-1 GagZip monocytes were differentiated into macrophages as previously described for THP-1 wild-type cells (Lund et al., 2016; Starr et al., 2018). Briefly, monocytes were seeded at 3.5×10^5^ cells/ml and incubated with 100 ng/ml phorbol 12-myristate 13-acetate (PMA) for 48 h. Then, non-adherent monocytes were removed, and macrophages were allowed to recover for 48 h in complete medium without PMA. After recovery, macrophages were HIV-1 induced or not (control) by the addition of 2 µg/ml doxycycline. Samples were collected 72 h post-induction unless otherwise indicated. All experiments were performed using THP-1 GagZip cells at three to seven passages after thawing.

### Macrophage fixation and immunostaining

THP-1 GagZip monocytes were differentiated into macrophages onto round cover glasses in 12-well plates. After HIV-1 induction or control conditions, macrophages were fixed by incubation with 4% paraformaldehyde (PFA) for 15 min. Then, cells were washed twice with D-PBS and free aldehyde groups were quenched by incubation with 0.1 M glycine for 10 min. The washing steps were repeated, and coverslips were kept in D-PBS at 4°C until staining.

For immunofluorescence staining, macrophages onto cover glasses were blocked and permeabilized for 45 min, by incubation with 1% BSA and 0.025% saponin diluted in D-PBS. Then, cover glasses were placed facing down onto 70 µl of a primary antibody mix and incubated in a hybridization oven for 1 h at 37°C. Then, samples were washed four times with D-PBS, 5 min each wash, and then placed facing down onto 70 µl of a secondary antibody mix and incubated as before in the hybridization oven. Each primary and secondary antibodies were used at 1:150 and 1:300 dilution, respectively, diluted in blocking or permeabilization solution. Later, samples were washed as before in D-PBS and incubated with DAPI for 10 min, rinsed, and air-dried while protected from direct light exposure. Finally, stained cover glasses were mounted onto clean glass slides using a drop of Shandon^TM^ Immu-mount, sealing the edges with regular nail polish. Mounted samples were stored at 4°C for at least one night before image acquisition. Incubations were performed at room temperature, except when indicated otherwise.

### Virus release quantification

HIV-1 Gag release was quantified from cleared supernatant of HIV-1-induced THP-1 GagZip macrophages by the ELISA HIV-1 p24 antigen capture assay (ABL Inc.), following the manufacturer's instructions. Briefly, virus particles in the supernatant were disrupted by incubation with the provided lysis solution for 1 h at 37°C. Then, wells were washed four times with 250 µl of D-PBS before adding 100 µl of the conjugated antibody solution and incubated for another hour at 37°C. The washing step was repeated and 100 µl of peroxidase substrate were added to each well, followed by 30 min incubation at room temperature in darkness. Finally, 100 µl of stop solution were added and absorbance at 450 nm was read within the next 10 min using an EnSpire® multimode plate reader.

When indicated, we evaluated virus release by a second approach, to discriminate between soluble and virus-integrated p24 following a previously established methodology to recover only assembled viruses (Waheed et al., 2009). In brief, 72 h after HIV-1 induction or control conditions, supernatants were cleared by centrifugation at 3000 ***g*** for 10 min, and then soluble proteins were removed by ultracentrifugation on top of a 20% sucrose cushion at 35,000 rpm, using rotor SW 55 Ti (Beckman Coulter) for 1 h at 4°C. Sedimented viruses were resuspended in 100 µl of D-PBS and stored at −20°C until use. Equal volumes (30 µl) from each ultracentrifuged sample were loaded into a 10% SDS-PAGE and evaluated for the presence of HIV-1 Gag–GFP and CD81 by western blotting. As treatments and control, this protocol was repeated upon addition of 1 µM PCLX-001 for 72 h to evaluate the effect on NMT1/2 inhibition on virus release, 5 µM MG132 for 24 h to evaluate proteasome-mediated degradation or 1% NP-40 for 30 min to disrupt membranes in the supernatant. Spike control was performed by adding 4 ml from DMSO HIV-1-induced sample to a PCLX-001-treated dish plate.

### Western blotting

To evaluate intracellular levels of protein expression, THP-1 GagZip macrophages were lysed with NP40 lysis buffer supplemented with protease inhibitor and incubation on ice for 30 min, vortexing every 10 min. Samples were centrifuged at 10,000 ***g*** for 10 min at 4°C to remove cell debris, and total amount of protein was quantified by Bradford reagent. 30 µg of total protein were heated at 95°C for 5 min and loaded into a 10 or 12% SDS-PAGE, performing electrophoresis at 100 V until the migration front reached the bottom part of the gel. In-gel samples were transferred to a nitrocellulose membrane at 250 mA for 3 h at 4°C. Then, membranes were blocked in 5% skim milk, diluted in Tris-buffered saline with 0.1% Tween 20 (TBS-T), for 45 min. Blocked membranes were incubated with primary and then the secondary antibodies, being washed three times between antibodies with 1× TBS-T for 10 min. All antibodies were incubated for 2 h or overnight at 4°C, with constant agitation. Primary antibodies were diluted in a solution of 1× TBS-T, 5% BSA and 0.02% sodium azide according to the manufacturer's recommendations. Dilutions used were: mouse anti-Arl8A/B, 1:250; mouse anti-CD81, 1:1000; sheep anti-p17, 1:1000; mouse anti-p24, 1:2500; rabbit anti-Rab7, 1:2500; mouse anti-β-actin, 1:5000, mouse anti-NMT1, 1:500; and mouse anti-NMT2, 1:500. Secondary antibodies were anti-rabbit-IgG, anti-mouse-IgG and anti-sheep-IgG antibodies coupled to horseradish peroxidase were all produced in donkeys and diluted 1:5000 in blocking solution. Incubations were performed at room temperature, except when indicated otherwise. Horseradish peroxidase signal was obtained by incubation with Western Lightning Pro chemiluminescent substrate and captured using a ChemiDoc Imaging system (Bio-Rad) using the chemiluminescence function, selecting ‘optimal autoexposure time’ and 2×2 pixel binning settings.

### Transmission electron microscopy

THP-1 GagZip monocytes were differentiated into macrophages onto an 8-well Lab-Tek Permanox chambered slide. At 72 h after HIV-1 induction, macrophages were washed twice with 0.1 M sodium cacodylate buffer (pH 7.2) and fixed overnight at 4°C with 2.5% glutaraldehyde diluted in wash solution. Fixed samples were washed three times with 0.1 M Na-cacodylate washing buffer for 20 min each and then incubated with 1% osmium tetroxide (Mecalab) for 1 h at 4°C. Then, samples were washed with ddH2O three times for 10 min before dehydration in a graded series of ethanol–deionized water solutions from 30 to 90%, 8 min each, followed by 100% ethanol twice for 10 min. Subsequently, samples were infiltrated with a mixture of Epon 812:ethanol in a ratio of 1:1 and then 3:1, each for 30 min, followed by 100% Epon 812 (Mecalab) for 1 h. Embedding was performed in the culture wells with new 100% Epon 812 and polymerized overnight at 60°C. Polymerized blocks were trimmed, and 100-nm ultrathin sections were cut with an UltraCut E ultramicrotome (Reichert Jung) and transferred onto 200-mesh copper grids (Electron Microscopy Sciences). Finally, samples were stained with 4% aqueous uranyl acetate (Electron Microscopy Sciences) for 8 min, and with Reynold's lead citrate (Thermo Fisher Scientific) for 5 min. Data were collected using a Tecnai 12 BioTwin 120 kV transmission electron microscope (TEM), equipped with a Gatan Ultrascan 4000 4k×4k CCD camera system, model 895.

### Extracellular Gag endocytosis

To evaluate whether THP-1 GagZip macrophages were able to form VCCs from endocytosed Gag present in the extracellular medium, we performed a spike experiment. THP-1 GagZip monocytes were differentiated into macrophages onto round cover glasses in 12-well plates and in a 10 cm dish at 3.5×10^5^ cells/ml. HIV-1 was induced during 72 h only in the 10 cm dish. Then, the supernatant was cleared from cell debris by centrifugation at 3000 ***g*** for 15 min and cleared supernatant from the HIV-1 induced sample was mixed in a 1:1 ratio with fresh RPMI. The diluted supernatant was used to replace the medium of of previously differentiated THP-1 GagZip macrophages in the 12-well plate. Every 24 h, samples were fixed by incubation with 4% PFA for 15 min. Then, cells were washed twice with D-PBS and free aldehyde groups were quenched by incubation with 0.1 M glycine for 10 min. The washing steps were repeated, and coverslips were kept in D-PBS at 4°C.

### Infection of human MDMs

Human macrophages were differentiated from monocytes isolated from PBMCs of healthy donors. Monocytes were isolated by negative selection, using the EasySep^TM^ human monocyte isolation kit (StemCell), following the manufacturer's instructions. Briefly, PBMC aliquots were thawed and washed with D-PBS supplemented with 2% FBS and 1 mM EDTA. Then, the cell suspension was stained with Trypan Blue, and the amount of living cells was determined. PBMCs were centrifuged at 500 ***g*** for 5 min and resuspended in supplemented D-PBS to give a concentration of 5×10^7^ cells/ml. The PBMC suspension was transferred to a 5 ml polystyrene round-bottom tube and isolation cocktail and platelet removal cocktail were added. After 5 min incubation, the magnetic beads were added, and samples were incubated for additional 5 min. Samples were placed in a magnetic rack and incubated for 3 min to allow separation of beads from monocytes. The monocyte-containing supernatant was transferred to a new tube and an aliquot was stained with Trypan Blue to assess the final number of isolated monocytes. Finally, monocytes were diluted in RPMI supplemented with 10% FBS and 50 ng/ml of granulocyte-macrophage colony-stimulating factor (GM-CSF) and seeded into 12-well plates at 5×10^5^ cells/ml. Monocytes were differentiated into macrophages for 7 days, with media replacements every 3 days.

To produce HIV-1 viral particles, HEK293T cells were seeded in 100 mm dishes and transfected with 10 µg of the HIV-1 BaL strain-coding plasmid. At 48 h post transfection, the supernatant was collected, cleared, and ultracentrifuged on top of a 20% sucrose cushion, at 28,000 rpm, using rotor Type 45 Ti Fixed-Angle (Beckman Coulter) for 1 h at 4°C, to concentrate assembled viral particles. Then, to infect macrophages, 5×10^5^ cells were inoculated with 10 ng of HIV-1 BaL (determined by p24 ELISA) and incubated for 48 h at 37°C. When indicated, macrophages were treated with 1 µM PCLX-001 (see below) at day 4 after infection. Samples were collected for immunofluorescence or western blotting at 4 or 7 days after infection.

### Enrichment of membrane organelles

To isolate membrane organelles from the cytoplasmic fraction, we followed previously described endosome purification protocol, with a few modifications (de Araujo et al., 2015; Lamberti et al., 2015). Briefly, THP-1 GagZip monocytes were differentiated into macrophages onto 150 mm dish plates. At 72 h after HIV-1 induction or in control conditions, macrophages were washed with cold D-PBS and detached using 10 ml of Accutase. Cells were transferred to a 15 ml conical tube and centrifugated at 500 ***g*** for 5 min. Cells were washed with D-PBS, transferred to 1.5 ml tubes, and centrifuged once again. Precipitated cells were resuspended in 750 µl of 0.1 µm filtered D-PBS. Cells were lysed mechanically by passing them through a 28 1/2-gauge needle attached to a 1 ml syringe about 20 times. Nuclear and mitochondrial fractions, along with non-lysed cells, were removed by centrifugation at 5000 ***g*** for 10 min at 4°C. The resultant cytoplasmic fraction was diluted four times in cold 3 mM imidazole (pH 7.4) 1 mM EDTA solution and ultracentrifuged at 35,000 rpm using SW 55 Ti Rotor (Beckman Coulter) for 2 h at 4°C. Precipitates were resuspended in 400 µl of 0.1 µm filtered D-PBS and stored in liquid nitrogen until staining. As a control, the same protocol was repeated using 750 µl of 0.1 µm filtered D-PBS alone.

### HIV-1 detection on endosomal populations by flow cytometry

Following the membrane organelles enrichment protocol, 30 µl aliquots were used for staining of different endosomal populations. Samples were incubated overnight at 4°C, in darkness, with an antibody mixture targeting early endosomes (EEA1–mFluorViolet 610), recycling endosomes (CD71–BrilliantViolet 421), late endosomes (Rab7–AlexaFluor 647), and lysosomes (Lamp1) using 1 µl of each per sample. To discriminate between intact and damaged LEL membranes, we used two different antibodies against Lamp1, one targeting the cytosolic tail (Lamp1–Cy3) and other one targeting the luminal side (Lamp1–PE/Cyanine7). Intact LELs were gated as cytosolic Lamp1^+^/luminal Lamp1^neg^, followed by cytosolic Lamp1^+^/ Rab7a^+^ signals. To determine background noise, the fluorescence minus one (FMO) control was performed for each antibody. Then, samples were diluted up to 220 µl in 0.1 µm filtered D-PBS, and unbound antibodies were removed following a washing protocol applied to small vesicle staining (Welsh et al., 2021). Briefly, 100 µl of CaptoCore 700 resin was washed twice with 0.1 µm filtered D-PBS allowing the resin to sink at room temperature for 30 min. The washed CaptoCore 700 resin was mixed with a stained sample, allowing the resin to sink at 4°C for 30 min. The top 200 µl were recovered and analyzed by flow cytometry using the ID7000 Spectral Cell Analyzer (Sony Biotechnology). Swarm detection control was performed by 2-fold dilution of each sample in a 96-well plate. Before sample acquisition, side and forward scatters were set up using the Apogee Mix beads (Apogee Flow Ltd.) to detect particles ranging from 0.11 to 1.3 µm in size. Acquisition was performed equally for all samples, recording all events for 2 min. To confirm the presence of membrane organelles, samples were disrupted with RIPA lysis buffer after staining. Data analyses and gating strategies were performed using FlowJo v10 software.

### Lysosomal staining in live samples

For LEL visualization in live-cell settings, macrophages were stained with LysoTracker Red DND-99 (Invitrogen), following the manufacturer's recommendations. Briefly, THP-1 GagZip monocytes were differentiated into macrophages onto a Lab-Tek®II chambered cover glass. 72 h after HIV-1 induction or control conditions, macrophages were washed with D-PBS and incubated in complete RPMI supplemented with 50 nM Lysotracker Red for 30 min at 37°C and 5% CO_2_. After incubation, cells were washed to remove excess dye. Samples were recorded immediately using a microscope with a top-stage incubator set up at 37°C and 5% CO_2_. For three-dimensional imaging, macrophages were recorded in several optical fields along the Z-axis with a confocal microscope. Events at the plasma membrane were recorded by total internal reflection fluorescence (TIRF) microscopy, set up at an imaging depth of 100 nm.

### Cathepsin B activity in live-cell imaging

To determine the degradative activity of lysosomes and their relationship with HIV-1 Gag, we stained them using the Cathepsin B assay kit (Magic Red), following the manufacturer's recommendations. Briefly, THP-1 GagZip monocytes were differentiated into macrophages in a 4-well Lab-Tek®II Chambered slide. 72 h after HIV-1 induction or control conditions, macrophages were washed with D-PBS and incubated with a 1:250 dilution of Magic Red dye during 30 min at 37°C. Magic Red is a cathepsin B substrate that will emit fluorescence when degraded. After incubation, samples were washed twice with D-PBS and replaced with fresh RPMI. The fluorescence signal was recorded during 20 min in several optical fields along the *Z*-axis with a confocal microscope, using an excitation laser at 594 nm.

### Arl8b modulation

At the time of HIV-1 induction or control conditions, THP-1 GagZip macrophages were transduced with lentivirus to overexpress Arl8b. After 72 h, cells were lysed or fixed as described above. Lentivirus particles were produced in HEK293T cells using a custom-made lentiviral plasmid (VectorBuilder) coding for the human *Arl8b* gene under the control of the cytomegalovirus promoter. Arl8b-coding lentivirus particles were produced by co-transfection of the lentiviral plasmid alongside the packaging plasmid psPAX2 (Addgene #12260) and the envelope plasmid pVSVg (Addgene #8454) in a ratio of 4:3:1, respectively. At 48 h post transfection, the supernatant was collected, cleared and stored at −80°C until use. Lentiviral transduction was carried out on THP-1 GagZip macrophages by adding equal parts of supplemented RPMI and supernatant containing Arl8b-coding lentiviruses. Samples were collected at 72 h after transduction.

For Arl8b knockdown, macrophages were transfected with the Dharmacon SMARTpool siRNA mixture, which contains four siRNA targeting *Arl8b* (siRNA sequences: 5′-GAUAGAAGCUUCCCGAAU-3′, 5′-CGUCAAUGUCAUCGCGUCA-3′, 5′-GAAGCAUGUGGGAGCGGUA-3′ and 5′-GAUAGAUGCUGCAGAUCGU-3′). The siRNA mixture was transfected using JetPrime following the manufacturer's instructions. Briefly, 5 µl of 5 µM SMARTpool siRNA (final concentration 25 nM) were mixed with 100 µl of JetPrime Buffer and 3 µl of JetPrime transfection reagent, followed by 10 min incubation at room temperature. Then, the transfection mixture was added onto THP-1 GagZip macrophages, and cells were incubated for 72 h at 37°C and 5% CO_2_.

### Macrophage-to-T cell transmission

To evaluate macrophage-to-T cell transmission, SupT1 CD4+ T cells were co-cultured with THP-1 GagZip macrophages following a previously reported protocol (Baxter et al., 2014), with slight modifications. Briefly, THP-1 GagZip monocytes were differentiated into 6-well plates, followed by *Arl8b* transduction as previously stated. At 48 h after HIV-1 induction, macrophages were washed and co-cultured with SupT1T cells at a concentration of 2.5×10^5^ cells/ml. As a control to discriminate between cell-to-cell and cell-free transmission, T cells were physically separated from macrophages by a polycarbonate membrane insert. At 24 h after co-culturing, SupT1 cells were recovered, centrifuged at 500 ***g*** for 5 min, and stained for viability by incubation with Fixable Viability Stain 780 (BD Horizon), washed with D-PBS, and fixed with 4% PFA for 20 min at room temperature. Then, the centrifugation step was repeated, and samples were resuspended in 200 µl of D-PBS. Transmitted HIV-1 to T cells was determined by Gag–GFP signal detection by flow cytometry, acquiring 15,000 events per sample in a BD LSR Fortessa™ Cell Analyzer. Data analyses and gating strategies were performed using FlowJo v10 software.

### N-myristoylation inhibition by PCLX-001

To prevent the myristoylation of HIV-1 Gag, we inhibited the NMT1/2 using the small-molecule PCLX-001 (also known as Zelenirstat or DDD86481), a first-in-class N-myristoylation inhibitor by Pacylex Pharmaceuticals (Sangha et al., 2022; Beauchamp et al., 2020; Sangha et al., 2024). At the time of HIV-1 induction or control conditions, macrophages were incubated in complete medium supplemented with different concentrations of PCLX-001 (from 0.1 to 2 µM), or an equivalent volume of DMSO as a vehicle control. At 72 h after co-treatments, samples were collected to evaluate intracellular and extracellular HIV-1 Gag protein levels, VCCs formation and HIV-1 Gag presence on LELs, as previously described. To evaluate the effectiveness of N-myristoylation inhibition, we used the myristoylation protein assay kit (Abcam), following the manufacturer's instructions. Briefly, 48 h after PCLX-001 treatment, cells were washed and incubated with fresh medium supplemented with an alkynyl-myristate analog. After 24 h, macrophages were washed with D-PBS and fixed for 15 min at room temperature with the provided fixative solution. Then, macrophages were permeabilized for 10 min and further incubated with the EZClick^TM^ Myristic Acid reaction solution followed by the DNA staining step. The reaction will add an azido-fluorophore to proteins that integrated alkynyl-myristate analog. The presence of labeled myristic acid into newly synthesized proteins was visualized by fluorescence microscopy, selecting the laser wavelength to detect DAPI and tdTomato signals.

### Cell viability

To determine cell viability after PCLX-001 treatment, THP-1 GagZip macrophages were seeded in a 6-well plate at 3.5×10^5^ cells/ml and differentiated to macrophages as above. At the time of HIV-1 induction, cells were treated with increasing concentrations of PCLX-001, ranging from 0.1 to 2 µM. After 72 h, macrophages were detached from the wells with Accutase and transferred to 5 ml round-bottom polystyrene test tubes for flow cytometry. Samples were washed with D-PBS and then centrifuged at 500 ***g*** for 5 min. Then, cells were resuspended in Fixable Viability Stain 780, diluted 1000 times in D-PBS, and incubated at room temperature for 20 min. After repeating the washing step, cells pellets were resuspended in 300 µl of 4% PFA, and fixed at room temperature during 10 min. A last wash in D-PBS was performed and cells were resuspended in 200 µl of 1X D-PBS. Viability staining signal was observed by flow cytometry, acquiring all events during 1 min per sample, using a BD LSR Fortessa™ Cell Analyzer. Data analyses and gating strategies were performed using FlowJo v10 software.

### Immunofluorescence image acquisition

For fixed samples, images were acquired using an inverted Zeiss LSM800 laser-scanning confocal microscope, equipped with an Airyscan GaAsP PMT detector. Samples were observed using oil-immersion 40× or 63× objectives, each of them with a numerical aperture of 1.40. Individual laser wavelength were defined using the ZEN 3.3 software ‘Smart setup’ function, choosing ‘Best signal’ to avoid leaking between channels. The lasers wavelength were selected according to the DAPI, eGFP, AlexaFluor-594 and AlexaFluor-647 emission spectrums. Image dimensions were set up for ‘optimal resolution’ depending on the selected objective, using a 16-bit depth and a 2× linewise averaging scan.

For live-cell experiments, recording was performed in an inverted Quorum Wave FX Spinning Disk confocal microscope, coupled with a Hamamatsu EM-CCD Digital Camera and a stage-top incubator set up at 37°C and 5% CO^2^. Live-cell movies were obtained using the oil-immersion 63× objective with a numerical aperture of 1.40. Gag–GFP and Lysotracker Red DND-99 signals were acquired with the diode lasers 488 nm and 561 nm, respectively, filtered at 525/50 nm for GFP, and 620/60 nm for Lysotracker Red. For three-dimensional acquisition, several optical fields on the *Z*-axis were acquired every 0.5 µm using the Velocity Software. During TIRF experiments, each laser was set up with an incident angle to excite only fluorophores that are up to 100 nm apart, between the cover glass and the basal side of cells, using the Metamorph software.

### Image analysis

All immunofluorescence images were analyzed in Imaris v10.0.1 software. Colocalization analyses were performed by manually setting the threshold signal for each channel based on control conditions. Colocalization was expressed as Manders' coefficient percentage (%) between two selected channels. For three-dimensional renditions, 3D to 2D XTension from Imaris was used to compile *Z*-stacks from the same field of view into two-dimensional images. To evaluate the motility of the particle, signals were rendered using the ‘Spots function’. Gag–GFP clusters (hence assumed to be VCCs) were rendered by setting up spots size at 0.4 µm, whereas LELs were rendered as spots of 0.5 µm in diameter. Gag–GFP spot tracks were determined using the Brownian motion algorithm and considering a maximum movement distance of 2 µm, with a gap of one frame. Separately, Lysotracker spot tracks were determined using the autoregressive motion algorithm, considering a maximum movement distance of 3.5 µm and a gap of one frame. Co-trafficking between Gag–GFP and Lysotracker spots was determined with the ‘spot-to-spot closest distance’ function, considering less than 0.1 µm distance between spot centers as colocalizing spots.

### Statistical analyses

All graphs were assembled and analyzed using GraphPad Prism v8.0.1. All graphs indicate the mean value of the data, with error bars indicating the s.e.m. from each dataset. For Manders’ colocalization analyses, graphs represent the average value obtained per picture frame; graphs from live-cell analyses represent the average value obtained per individual cell. A two-tailed unpaired *t*-test with Welch's correction or an one-way ANOVA were performed for statistical analyses. For ANOVA, multiple comparisons were performed using Dunnett's test to compare against a control condition, or Tukey's test to compare all datasets with each other. Statistical tests are specified in each figure legend. Asterisk symbols represent statistical differences based on *P*-values as follows: *P≤0.05; **P≤0.01; ***P≤0.001; ****P≤0.0001.

## Supplementary Material

10.1242/joces.263588_sup1 Supplementary information
