# Supplementary material for: HIV-1 N-myristoylation-dependent hijacking of late endosomes/lysosomes to drive Gag assembly in macrophages
Source: J Cell Sci. 2024 Nov 21;137(22):jcs263588. doi: 10.1242/jcs.263588 (PMC11607699; doi:10.1242/jcs.263588)
Supplement: Supplementary information [file joces-137-263588-s1.pdf]

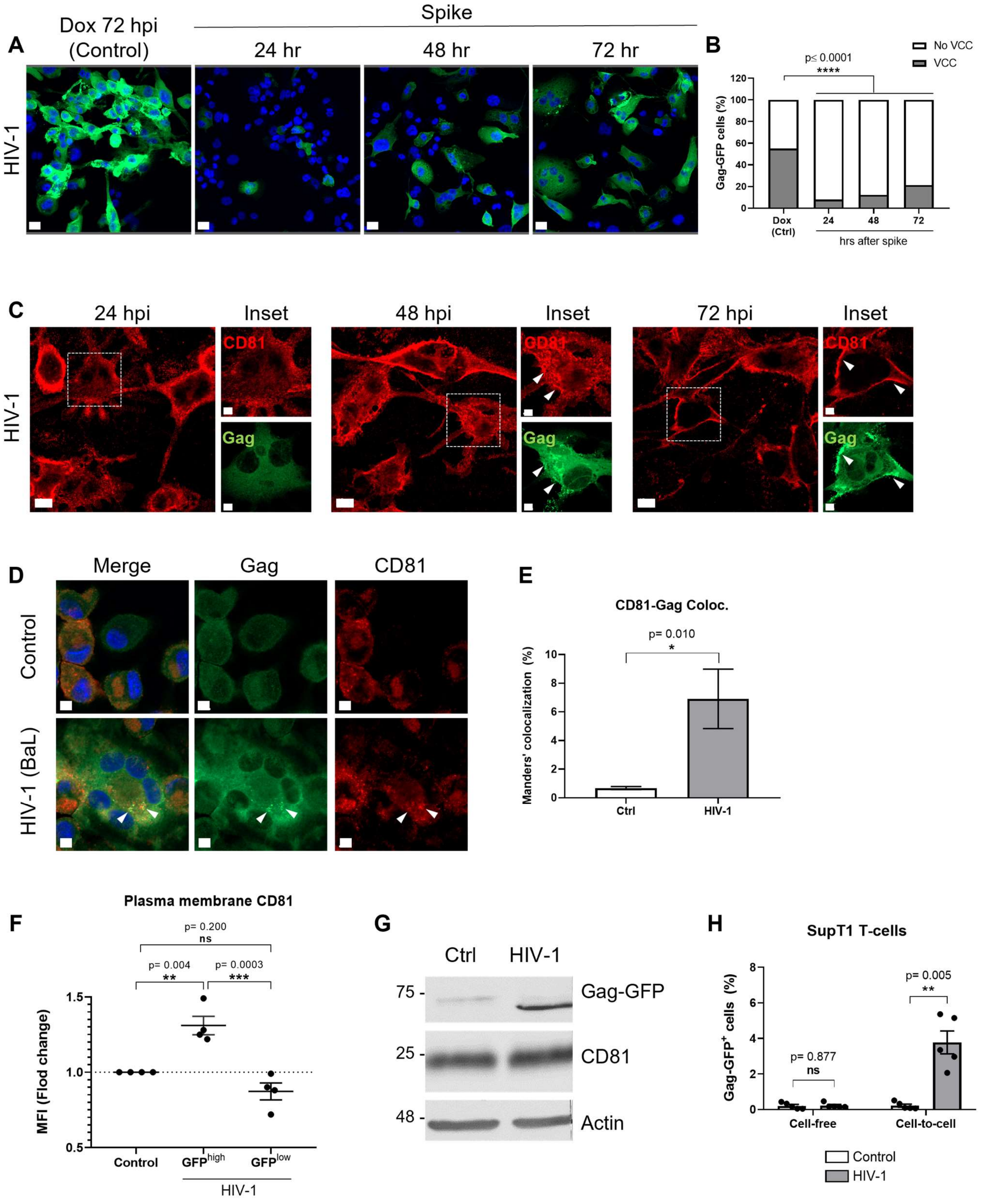

Journal of Cell Science • Supplementary information

Fig. S1. THP-1 GagZip macrophages resembles HIV-1 infection as in human macrophages.

(A) Confocal microscopy from fixed THP-1 GagZip macrophages 72 hrs after HIV-1 induction, or non-induced but spiked with supernatant from induced samples. Cells are labeled with DAPI (blue) and Gag- GFP (green). Size bars represent 20 µm.

(B) From A. Percentage of Gag-GFP expressing macrophages that have GFP clusters, from 72 hrs HIV-1 induced macrophages (Dox Ctrl), or after the viral spike during 24, 48 or 72 hrs.

(C) Confocal microscopy from fixed THP-1 GagZip macrophages collected every 24 hrs after HIV-1 induction. Each panel represents a timepoint with a respective enlarged area (inset), showing Gag-GFP (green) and CD81 (red). White arrows indicate the colocalization between Gag-GFP and CD81. Size bars represent 15 µm and 5 µm (insets).

(D) Confocal microscopy from fixed human MDMs uninfected (control) or infected with HIV-1 BaL for 4 days. Samples were stained by immunofluorescence to detect Gag (green) and CD81 (red). White arrows indicate colocalization between Gag and CD81 clusters. Size bars represent 5 µm.

(E) From (D). Bar graph representing Manders' percentage of colocalization of CD81 over Gag-GFP, evaluated at 4 days after HIV-1 BaL infection. Uninfected condition labeled as Ctrl.

(F) Endogenous CD81 MFI levels at the plasma membrane, quantified by flow cytometry in non-permeabilized THP-1 GagZip macrophages, 72 hrs after HIV-1 induction or control conditions. HIV-1 induced macrophages were sorted based on their Gag-GFP levels of expression. Data was normalized against control conditions.

(G) Western blot from THP-1 GagZip macrophages 72 h after HIV-1 induction or control conditions. Membrane was evaluated for Gag-GFP and endogenous CD81 expression levels. ß-actin was used as a loading control.

(H) Bar graph representing the percentage of SupT1 T cells expressing HIV-1 Gag-GFP$^+$ after 24 hrs of co-culture with THP-1 GagZip macrophages. T cells were incubated with macrophages at 48 hrs after HIV-1 induction or control conditions. T cells were physically separated from macrophages by a cell- culture insert or not, to discriminate between cell-free and cell-to-cell viral transmission, respectively. Data representative from 2 independent experiments. Graphs are presented as mean ± SEM. Statistical analysis in (B) and (F) are One-way ANOVA, with Dunnett's post hoc test to compare each data set against Dox (Ctrl) condition (B), and Tukey's post hoc test to compare between all data sets (F). For (E) and (H), unpaired T-test with Welch's correction. * P 0.05; ** P 0.01; *** P 0.001; **** P 0.0001.

Journal of Cell Science • Supplementary information

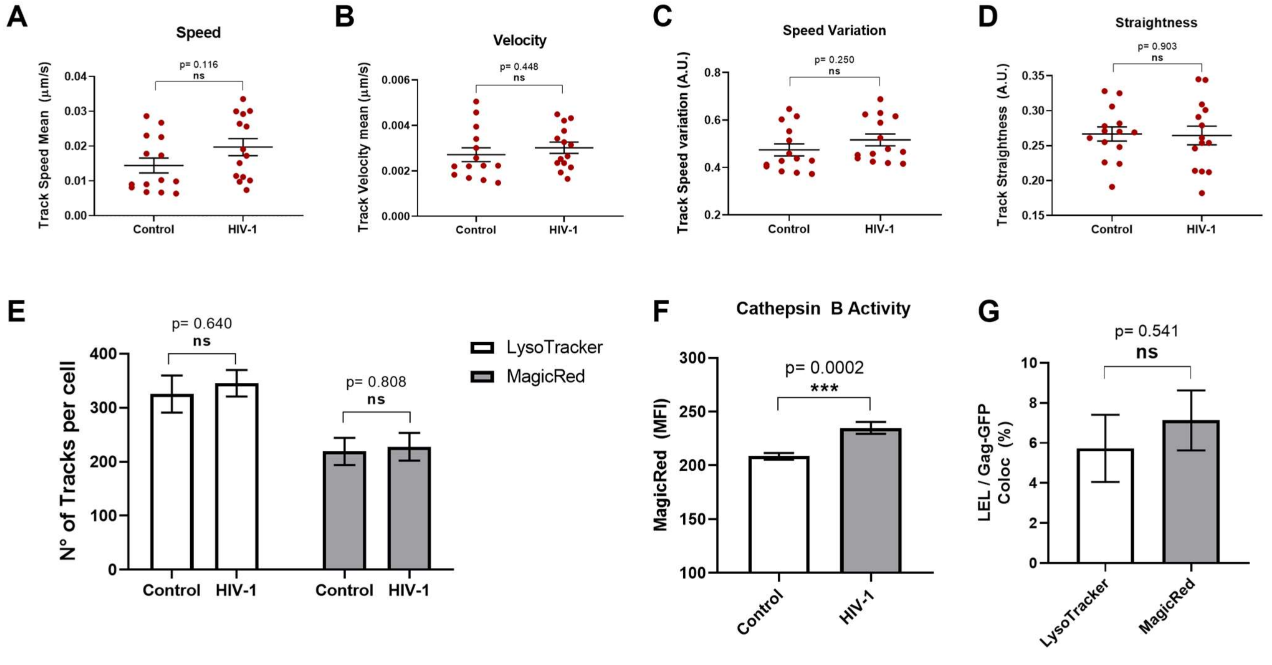

Fig. S2. HIV-1 induction in THP-1 GagZip macrophages does not alter the overall LEL motility nor biogenesis.

(A to D) Single molecule track analysis from Fig. 4 before sorting between colocalizing and non-colocalizing LEL with HIV-1 Gag-GFP. LEL spots were rendered and tracked in Imaris v10.0.0 software. Motility parameters were analyzed 72 h after HIV-1 induction or control conditions, determining LEL's mean tracks speed (A), velocity (B), speed variation (C) and straightness (D). Each dot represents the mean value obtained per one cell.

(E) Number of detected LEL per THP-1 GagZip macrophage by two different dyes, 72 h after HIV-1 induction or control conditions. LysoTracker Red to detect the whole LEL population, and Magic Red to detect only LEL with active Cathepsin B.

(F) Percentage of colocalizing Gag-GFP with LEL from THP-1 GagZip macrophages 72 hrs after HIV-1 induction. LEL were detected with LysoTracker Red or Magic Red.

(G) Cathepsin B activity measured as Magic Red MFI. 72 h after HIV-1 induction or control conditions. THP-1 GagZip macrophages were incubated with Magic Red for 30 mins at 37° before analyses.

Data representative from 2 (Magic Red) or 4 (Lysotracker) independent experiments. Graphs are presented as mean ± SEM. Statistical analysis in all graphs are unpaired T-test with Welch's correction. *** P < 0.001.

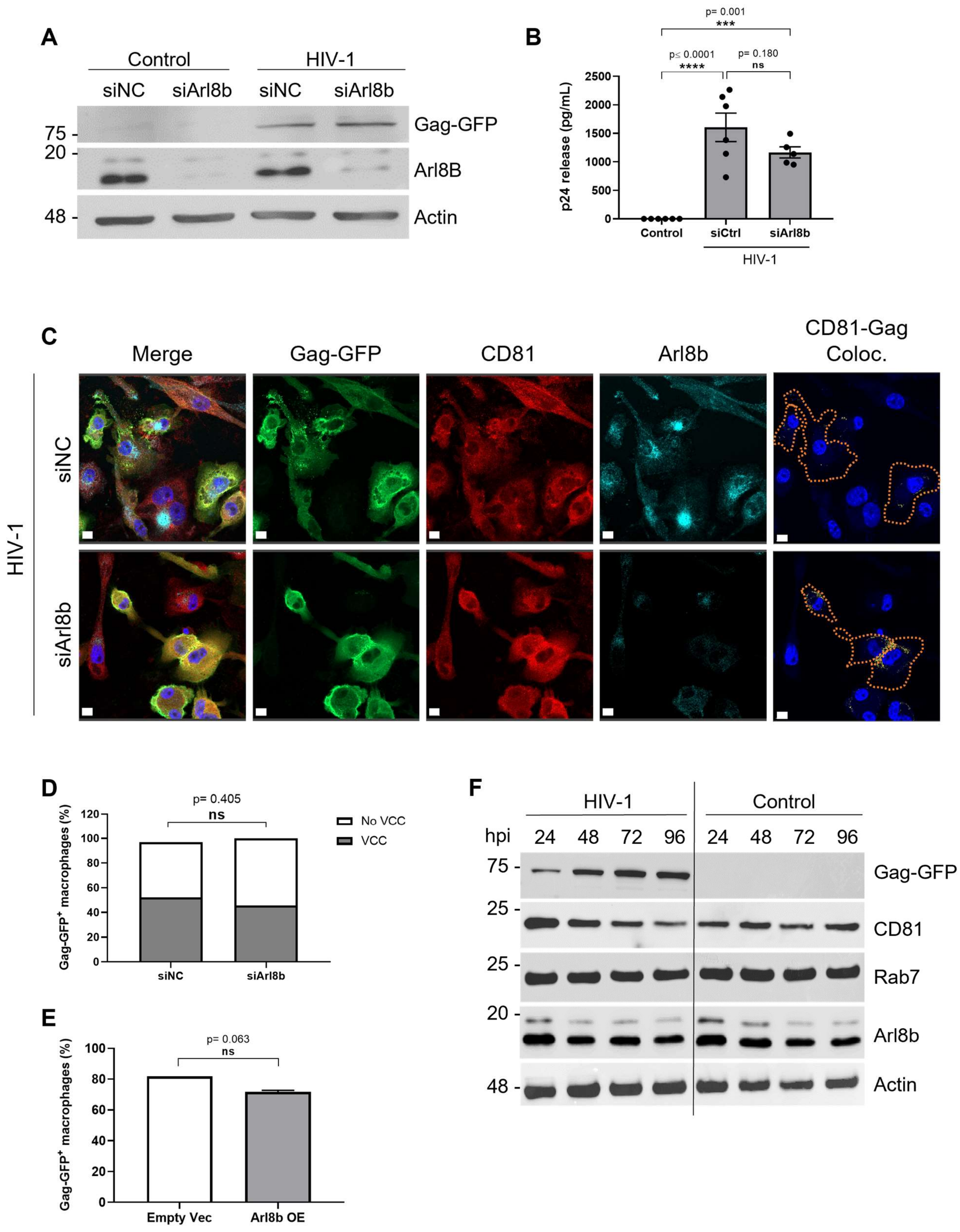

Journal of Cell Science • Supplementary information

Fig. S3. Arl8b downregulation does not affect VCCs formation.

(A) Western blot after simultaneous HIV-1 induction (or control conditions) and transfection with siRNA targeting Arl8b or a negative control siRNA (siNC). 72 h after co-treatments, cells were lysated and evaluated for HIV-1 Gag-GFP and Arl8b expression, using ß-actin as a loading control.

(B) HIV-1 p24 release into the supernatant measured by ELISA. THP-1 GagZip macrophages' supernatants were collected 72 h after simultaneous HIV-1 induction (or control conditions), and transfection with siRNA targeting Arl8b or a negative control siRNA (siNC)

(C) Confocal microscopy from fixed THP-1 GagZip macrophages evaluated for Gag-GFP expression (green), the VCCs marker CD81 (red), and Arl8b expression (cyan) by immunofluorescence, 72 h after simultaneous HIV-1 induction (or control conditions), and transfection with siRNA targeting Arl8b or a negative control siRNA (siNC). Cells with VCCs, identified as colocalizing signal between Gag-GFP and CD81 (yellow signal), are represented on the right-end panel. Cells' perimeters are delineated with orange if cells have VCCs, and white otherwise. Size bars represent 10 µm.

(D) From (C). Percentage of macrophages showing VCCs phenotype, defined by the colocalization between Gag-GFP and CD81. Gag-GFP expressing cells were counted based on Arl8b downregulation, or transfection with negative control siRNA (siNC).

(E) Related to Fig. 5. Percentage of THP-1 GagZip macrophages that express Gag-GFP 72 hrs after simultaneous HIV-1 induction and transduction with the Arl8b-coding or empty vector.

(F) Western Blot from THP-1 GagZip macrophages cell lysates collected every 24 h after HIV-1 induction or control conditions. Membranes were evaluated for Gag-GFP expression and the endogenous levels of CD81, Rab7 and Arl8b, using ß-actin as a loading control.

Data representative from 2 independent experiments. Graphs are presented as mean ± SEM. Statistical analysis in (B) is One-way ANOVA, using Tukey's post hoc test to compare between all data sets. In (D) and (E), unpaired T-test with Welch's correction. *** $P < 0.001$; **** $P < 0.0001$.

Journal of Cell Science • Supplementary information

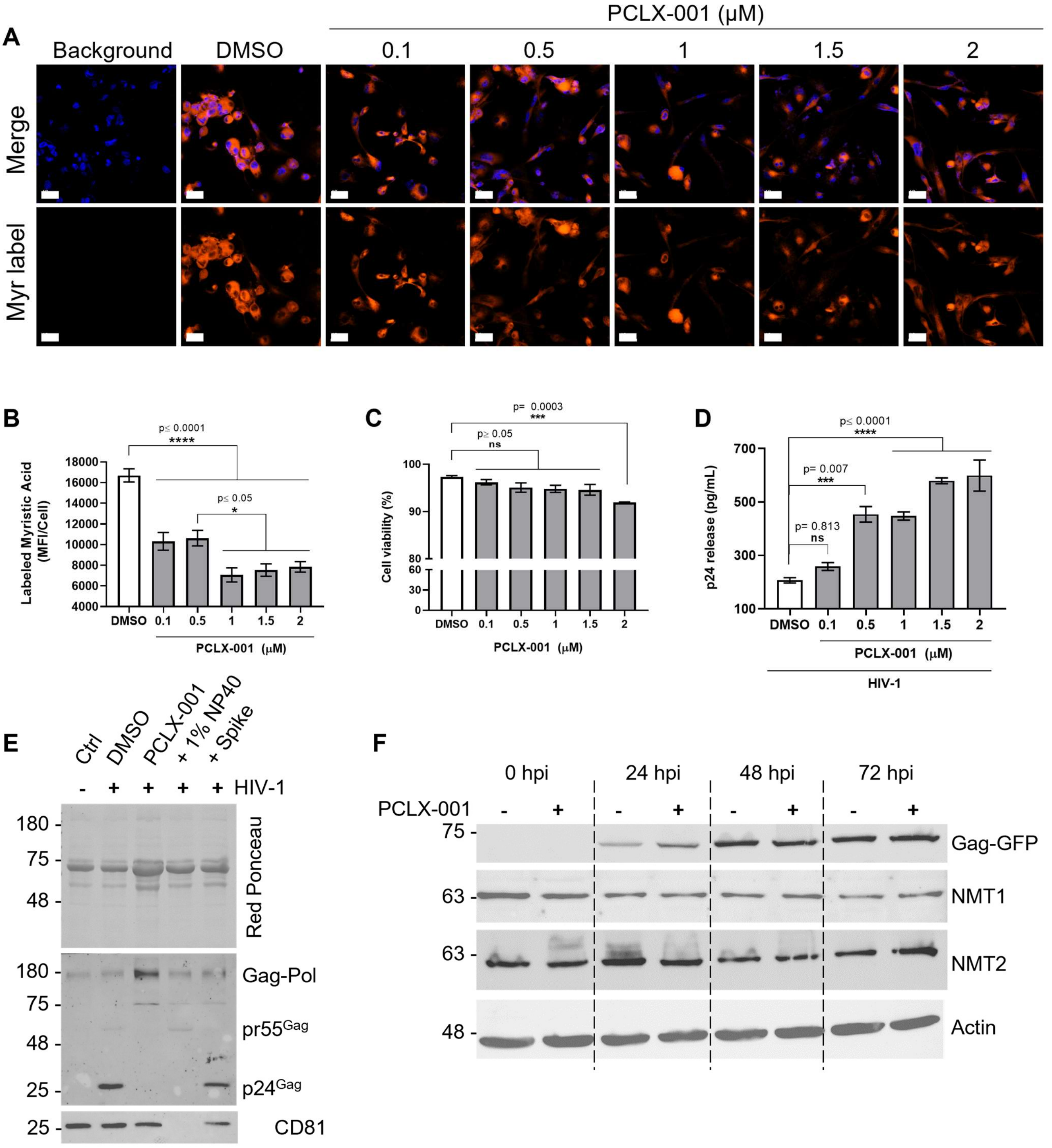

Fig. S4. PCLX-001 inhibits proteins myristoylation without affecting NMT proteins expression levels.

(A) Confocal microscopy from fixed THP-1 GagZip macrophages evaluated for myristic acid integration by immunofluorescence. Macrophages were incubated with DMSO or increasing concentrations of PCLX-001. Labeled myristic acid (red) was observed 72 h after treatment. Top panel depicts the merged channels with DAPI for nuclear staining. Size bars represent 40 µm.

Journal of Cell Science • Supplementary information

(B) From (A). MFI from labeled myristic acid in THP-1 GagZip macrophages after treatment with DMSO or increasing concentrations of PCLX-001.

(C) THP-1 GagZip macrophages viability 72 hrs after treatment with DMSO or increasing concentrations of PCLX-001.

(D) HIV-1 p24 release into the supernatant measured by ELISA. THP-1 GagZip macrophages' supernatants were collected 72 h after simultaneous HIV-1 induction, and DMSO or increasing doses of PCLX-001.

(E) Western blot from HIV-1 transfected HeLa cells to evaluate viral assembly by using concentrated viral particles from the supernatant 48 hrs after transfection, treated with DMSO or 1 µM PCLX-001. Red Ponceau staining was used as a loading control, and membrane was incubated with anti-HIV-1 p24 or anti-CD81.

(F) Western Blot from THP-1 GagZip macrophages samples collected every 24 h after simultaneous HIV-1 induction, and treatment with 1 µM PCLX-001 or DMSO. The membrane was evaluated to detect Gag-GFP, NMT1 and NMT2 expression levels, using ß-actin as a loading control.

Data representative from 2 independent experiments. Graphs are presented as mean ± SEM. Statistical analysis from (B), (C), and (D) are One-way ANOVA, using Tukey's post hoc test to compare between all data sets (B), or Dunnett's post hoc test to compare each data set against DMSO condition (C) and (D). * $P < 0.05$; *** $P < 0.001$; **** $P < 0.0001$.

Journal of Cell Science • Supplementary information

Table S1. Reagents and kits

Reagent Name	Brand	Cat #
**Cell Culture**		
1cc U-100 insulin syringe 28G ½	BD	329424
2-Mercaptoethanol	Gibco	21985-023
96 well, black/Clear Tissue Culture Plate	BD Falcon	353219
Accutase	StemCell	07920
Cell culture insert. 3.0 µm Polycarbonate Membrane	Nunc	140642
Cell Lifter	Biologix	70-2180
Doxycycline	Sigma	D9891
D-PBS	Multicell	311-425-CL
Dulbecco's Modified Eagle Medium	Gibco	11965-092
Granulocyte-macrophage colony-stimulating factor	PeproTech	300-03
Heat Inactivated FBS	Gibco	12484-028
JetPRIME®	Polyplus	101000046
Lab-Tek®II Chambered #1.5 Coverglass system	Nunc	155382
Lab-Tek® Permanox® Chambered Slide	Nunc	177445
LysoTracker™ Red DND-99	Invitrogen	L7528
ON-TARGETplus SMART pool siRNA human Arl8b	Dharmacon	L-020294-01-0005
Negative Control siRNA	Qiagen	1027310
PCLX-001	Pacylex Pharmaceuticals	N.A.
Penicillin-Streptomycin-Glutamine Solution	Multicell	450-202-EL
Phorbol 12-myristate 13-acetate (PMA)	Sigma	P8139
PhosSTOP	Roche	04 906 837 001
pLV-Puro-CMV>hArl8b	VectorBuilder	VB211015-1321esz
Protease inhibitor Cocktail	VWR	M221-1ML
PureFlow Sterile syringe filter, pore size 0.10 µm	Globe Scientific Inc.	SF-PES-1030-S

Journal of Cell Science • Supplementary information

RPMI Medium 1640	Gibco	11875-093
TC Dish 150 mm, Standard	Sarstedt	83.3903
TC Plate 12-well, Standard	Sarstedt	83.3921
TC Plate 48-well, Standard	Sarstedt	83.3923
TC Plate 6-well, Standard	Sarstedt	83.3920
Trypsin-EDTA Solution	Sigma	T4049
Ultracentrifuge tubes	Beckman Coulter	326819
**Immunofluorescence / Flow Cytometry**		
5 mL Polystyrene Round-Bottom Tube	Falcon	352054
ApogeeMix beads	Apogee	1527
Blocker™ BSA (10%) in PBS	Thermo Scientific	37525
CapTO^{tm} Core 700	Cytiva	17548101
Circular 18 mm Ø cover glasses, 0.15 mm thickness	VWR	16004-300
Circular 12 mm Ø cover glasses, 0.15 mm thickness	Thermo Scientific	12-545-80
DAPI (4',6-Diamidino-2-Phenylindole, Dihydrochloride)	Invitrogen	D1306
Donkey, anti-mouse IgG AlexaFluor™ 594	Invitrogen	A21203
Donkey, anti-mouse IgG AlexaFluor™ 647	Invitrogen	A31571
Donkey, anti-Rabbit IgG AlexaFluor™ 594	Invitrogen	A21207
Donkey, anti-Rabbit IgG AlexaFluor™ 647	Invitrogen	A31573
Fixable Viability Stain 780	BD Horizon	565388
Goat, anti-mouse IgG HP	Cell Signaling	7076S
Goat, anti-Rabbit IgG HP	R&D Systems	HAF008
Mouse, anti-Arl8 a/b	Santa Cruz Biotechnology	sc-398635
Mouse, anti-CD81	Abcam	ab59477
Mouse, anti-EEA1 mFluor Violet 610 SE	Novus	NBP2-36568MFV610
Mouse, anti-human CD107a (Lamp1) PE/Cyanine7	BioLegend	328618
Mouse, anti-human CD71 Brillant Violet 421™	BioLegend	334121

Journal of Cell Science • Supplementary information

Mouse, anti-NMT1	ProteinTech	67984-1-Ig
Mouse, anti-NMT2	Novus	NBP2-01676
Mouse, anti-p24	HIV reagent program (NIH)	3537
Mouse, anti-ß-Actin	Invitrogen	AM4302
Rabbit, anti-CD81	Cell Signaling	52892S
Rabbit, anti-Lamp1	Cell Signaling	9091S
Rabbit, anti-Lamp1 Cy3® (Cytosolic tail)	Abcam	ab67283
Rabbit, anti-Rab7	Abcam	ab137029
Rabbit, anti-Rab7 AlexaFluor™ 647	Abcam	ab198337
Shandon™ Immu-Mount™	Thermo Scientific	9990402
Sheep, anti-p17	HIV reagent program (NIH)	286 (discontinued)
**Commercial Kits**		
Bio-Rad Protein Assay Dye Reagent	Bio-Rad	5000006
Cathepsin B Activity Assay Kit (Magic Red)	Abcam	ab270772
EasySep™ Human Monocyte Isolation Kit	Stemcell	19359
E.Z.N.A.® Plasmid DNA Maxi Kit	Omega Bio-Tek	D6922-02
HIV-1 p24 Antigen Capture Assay	ABL inc.	5421
Myristoylated Protein Assay Kit (Red)	Abcam	Ab2732270
Western Lightning® ECL Pro	PerkinElmer	NEL121001EA
**Salts / Others**		
Acrylamide/Bis-Acrylamide (37.5:1) 40% Solution	BioShop	ACR005.500
Ammonium persulfate (APS)	BioShop	AMP001.100
Cycloheximide	Calbiochem	239763
Dimethyl Sulfoxide (DMSO)	BioShop	DMS666.100
EDTA	BioShop	EDT001.1

Journal of Cell Science • Supplementary information

Glycine	BioShop	GLN001.10
Imidazole	Sigma-Aldrich	I2399-100G
MG-132	Sigma-Aldrich	M7449-1ML
Nitrocellulose membranes 0.45 µm	Bio-Rad	1620115
NP-40 Igepal® CA-630	Sigma-Aldrich	I3021-100ML
OmniPur® Polyoxyethylene (Tween 20)	Millipore Sigma	9480
Paraformaldehyde (PFA)	Sigma-Aldrich	P6148-500G
Potassium chloride (KCl)	Fisher BioReagents	BP366-500
RIPA Buffer	Sigma-Aldrich	R0278-500ML
Saponin	Sigma	47036-50G-F
Sodium chloride (NaCl)	EMD	SX0420-5
Sodium Dodecyl Sulfate (SDS)	Multicell	800-100-CG
Sucrose	BioShop	SUC507.5
Tetramethyl ethylenediamine (TEMED)	Bio-Rad	1610801
Tris	BioShop	TRS0015

Journal of Cell Science • Supplementary information

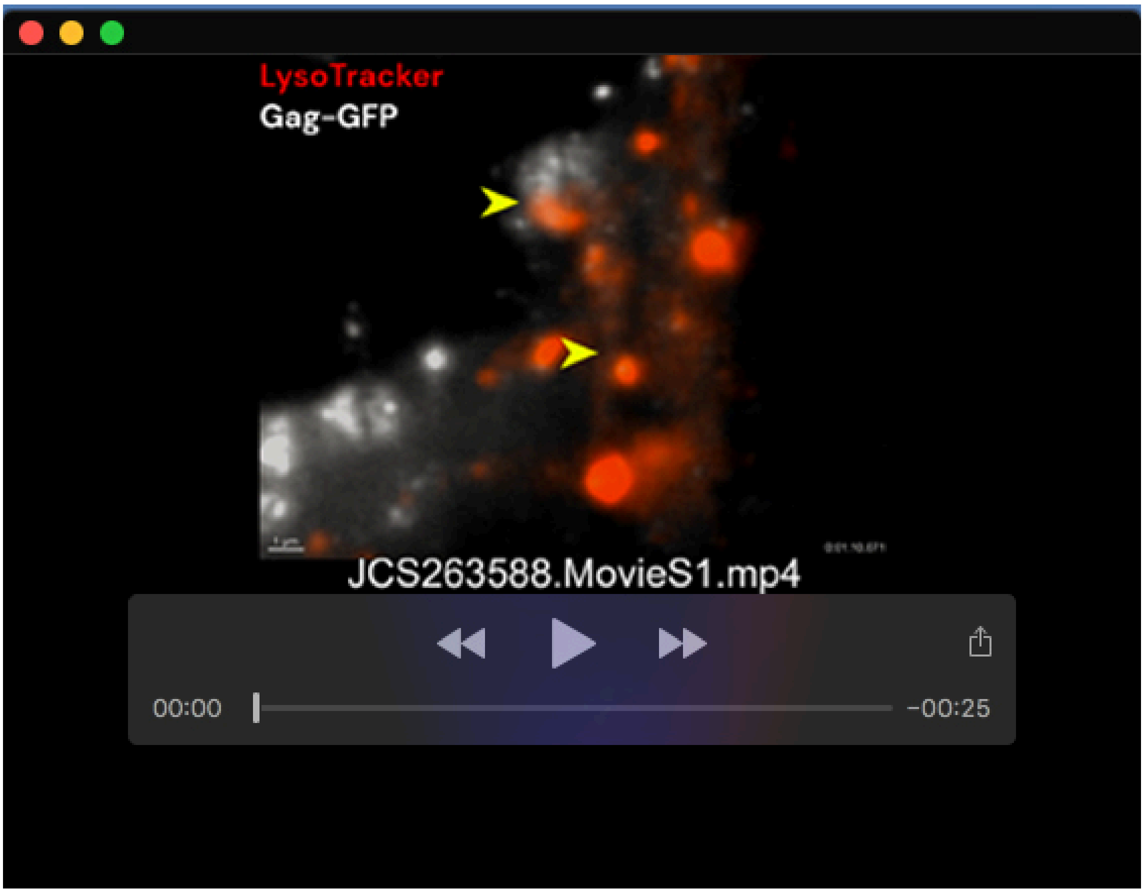

Movie 1. LEL colocalize with HIV-1 Gag at the plasma membrane of macrophages.

Total Internal Reflection Fluorescence (TIRF) Microscopy from THP-1 GagZip macrophages 72 h after HIV-1 induction and incubation with 50 nM Lysotracker red. The movie shows a 10 min recording of LEL (red) in close proximity to Gag-GFP (white) clusters. Size bar represents 1 µm. The movie was processed and assembled using Imaris v10.0.0.

Journal of Cell Science • Supplementary information

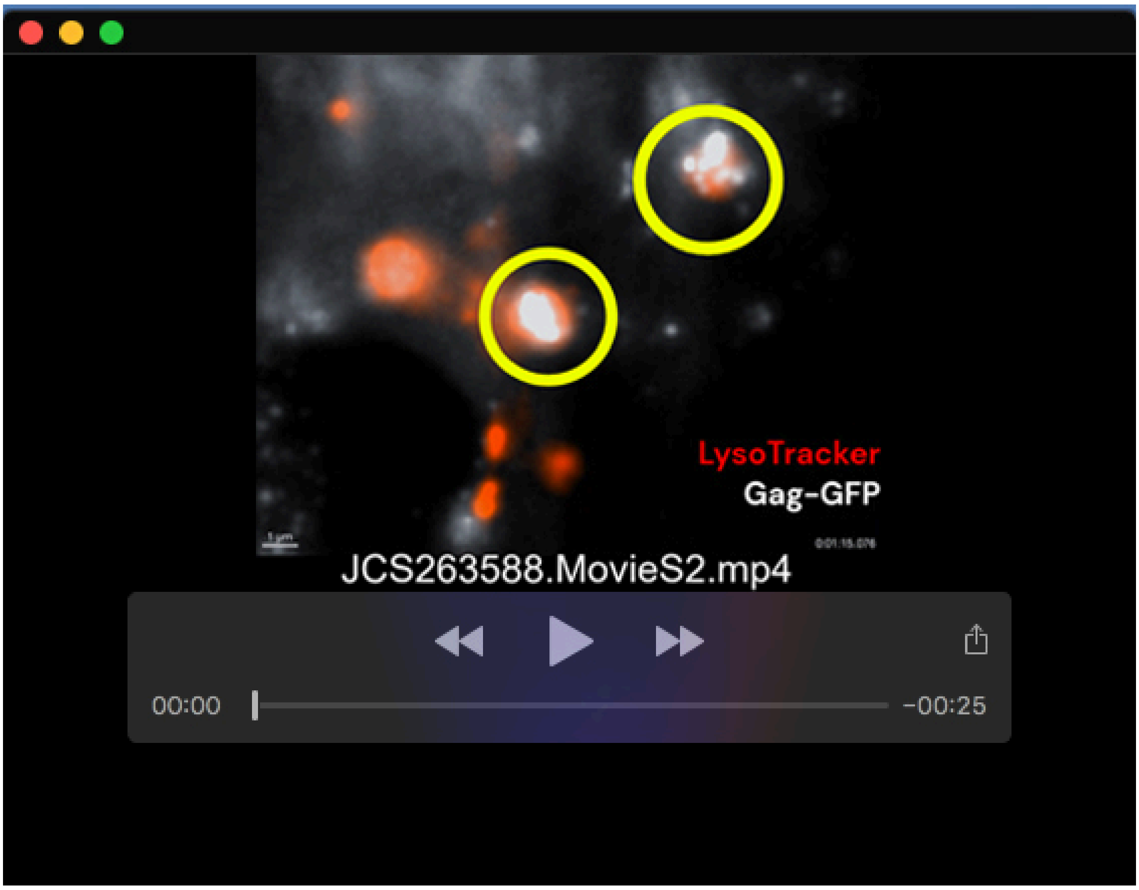

Movie 2. LEL close to the plasma membrane are decorated with HIV-1 Gag.

Total Internal Reflection Fluorescence (TIRF) Microscopy from THP-1 GagZip macrophages 72 h after HIV-1 induction and incubation with 50 nM Lysotracker red. The movie shows a 10 min recording of big LEL (red) that are decorated with several Gag-GFP (white) molecules, showing a static movement near the plasma membrane. Size bar represents 1 µm. The movie was processed and assembled using Imaris v10.0.0.

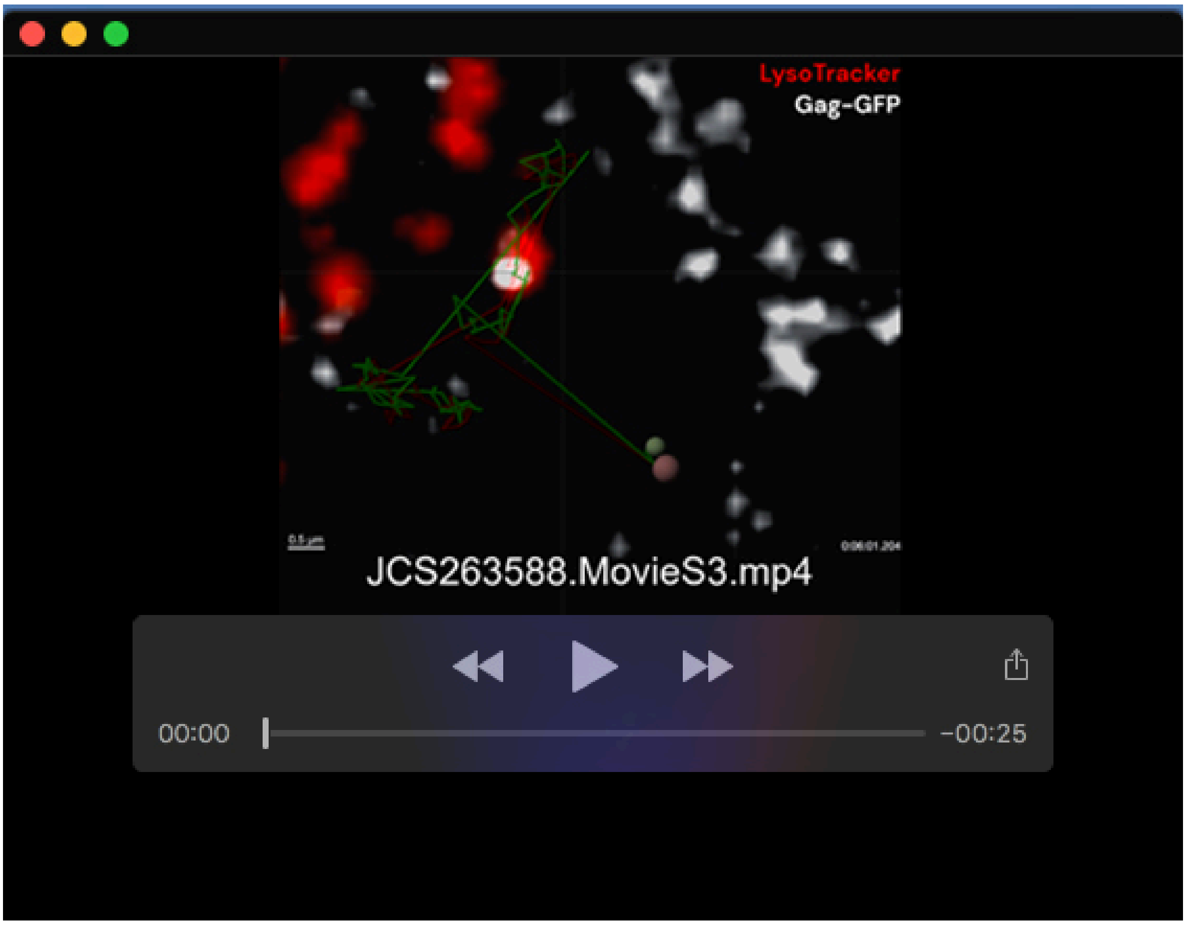

Movie 3. LEL co-traffic with HIV-1 Gag for extended periods of time.

Live-cell imaging 2D rendition obtained from recording and stacking several images from the Z-axis in a confocal microscope using THP-1 GagZip macrophages 72 h after HIV-1 induction and incubation with 50 nM Lysotracker red. The movie shows a 30 min recording of a LEL (red) that co-traffics with Gag- GFP (white) during the entire length of the movie. Size bar represents 0.5 µm. The movie was processed and assembled using Imaris v10.0.0.

Journal of Cell Science • Supplementary information

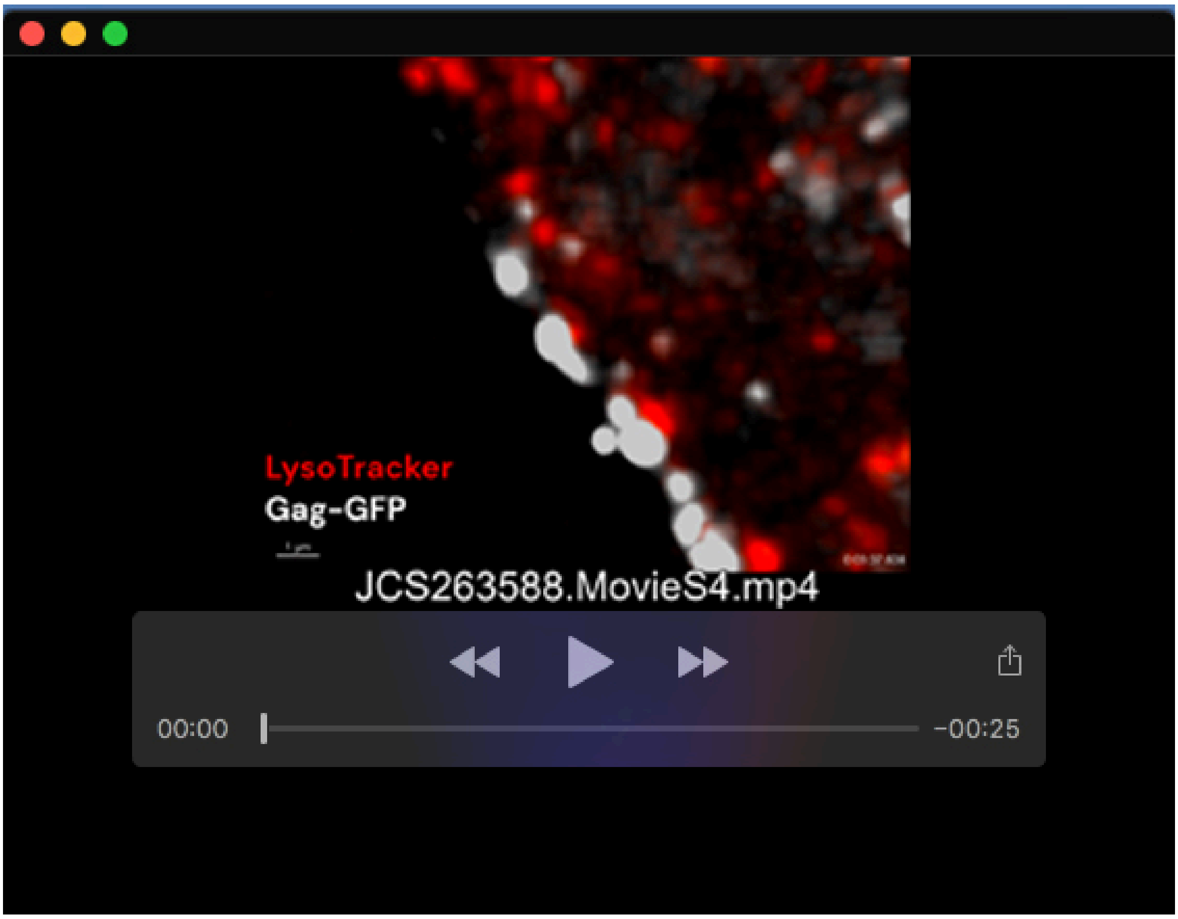

Movie 4. HIV-1 Gag-carrying LEL come together at VCCs.

Live-cell imaging 2D rendition obtained from recording and stacking several images from the Z-axis in a confocal microscope using THP-1 GagZip macrophages 72 h after HIV-1 induction and incubation with 50 nM Lysotracker red. The movie shows a 15 min recording of a group of LEL (red) that co-traffics with Gag-GFP (white) molecules and deliver them as cargo to already formed Gag-GFP clusters. Size bar represents 1 µm. The movie was processed and assembled using Imaris v10.0.0.

Journal of Cell Science • Supplementary information
